# Wnt co-receptors Lrp5 and Lrp6 differentially mediate Wnt3a signaling in osteoblasts

**DOI:** 10.1371/journal.pone.0188264

**Published:** 2017-11-27

**Authors:** Aimy Sebastian, Nicholas R. Hum, Deepa K. Murugesh, Sarah Hatsell, Aris N. Economides, Gabriela G. Loots

**Affiliations:** 1 Lawrence Livermore National Laboratories, Physical and Life Sciences Directorate, Livermore, CA, United States of America; 2 UC Merced, School of Natural Sciences, Merced, CA, United States of America; 3 Regeneron Pharmaceuticals, Tarrytown, NY, United States of America; Kyungpook National University School of Medicine, REPUBLIC OF KOREA

## Abstract

Wnt3a is a major regulator of bone metabolism however, very few of its target genes are known in bone. Wnt3a preferentially signals through transmembrane receptors Frizzled and co-receptors Lrp5/6 to activate the canonical signaling pathway. Previous studies have shown that the canonical Wnt co-receptors Lrp5 and Lrp6 also play an essential role in normal postnatal bone homeostasis, yet, very little is known about specific contributions by these co-receptors in Wnt3a-dependent signaling. We used high-throughput sequencing technology to identify target genes regulated by Wnt3a in osteoblasts and to elucidate the role of Lrp5 and Lrp6 in mediating Wnt3a signaling. Our study identified 782 genes regulated by Wnt3a in primary calvarial osteoblasts. Wnt3a up-regulated the expression of several key regulators of osteoblast proliferation/ early stages of differentiation while inhibiting genes expressed in later stages of osteoblastogenesis. We also found that Lrp6 is the key mediator of Wnt3a signaling in osteoblasts and Lrp5 played a less significant role in mediating Wnt3a signaling.

## Introduction

Wnt proteins constitute a family of 19 highly conserved secreted signaling proteins that have various roles in development and disease. Wnts activate either β-catenin dependent canonical signaling pathway or β-catenin independent non-canonical pathways to perform their diverse functions [1]. Several studies have identified Wnt signaling pathway as a key regulator of bone development and homeostasis. Canonical Wnt signaling has been shown to promote osteoblast differentiation, enhance osteoblast proliferation, maturation and mineralization, and inhibit osteoblast apoptosis [2]. Canonical Wnt signaling has also been shown to inhibit osteoclast differentiation [3]. Several Wnt ligands including Wnt1, Wnt3a and Wnt10b preferentially activate canonical Wnt signaling [4]. Previous studies have shown that these Wnts promote bone formation [5–9]. However, very little is known about the molecular mechanism by which canonical Wnts regulate osteoblastogenesis and bone formation or target genes regulated by these Wnts during this process.

Wnt proteins activate canonical signaling by binding to transmembrane receptors Frizzled and co-receptors Lrp5/6 which trigger a cascade of intracellular events that facilitate the translocation of β-catenin to the nucleus where it interacts with Tcf/Lef family of transcription factors and activates transcription of Wnt-responsive genes [10]. Previous studies have suggested that Wnt co-receptors Lrp5 and Lrp6 have both overlapping and non-redundant functions in the skeleton [11, 12]. Mice lacking *Lrp6* display severe developmental defects and die shortly after birth [13] whereas *Lrp5* knockout mice have no developmental defects but acquire a low bone mass phenotype postnatally [14]. In humans, mutations in *Lrp5* result in high bone mass (HBM) or low bone mass (LBM) depending on the nature of the mutation [15–17]. Mice lacking either *Lrp5* or *Lrp6* in mature osteoblasts displayed LBM and mice lacking both *Lrp5* and *Lrp6* in osteoblasts developed severe osteopenia [11]. Also, mechanical loading induced bone formation was significantly reduced in *Lrp5* knockout mice whereas mice harboring HBM-causing *Lrp5* mutations exhibited increased bone formation in response to loading [18, 19]. Wnt-Lrp5 signaling has also been shown to regulate fatty acid metabolism in the osteoblast [12]. These findings suggest that both Lrp5 and Lrp6 are essential for normal postnatal bone homeostasis [12, 14]. However, very little is known about their specific roles in mediating canonical Wnt signaling in osteoblasts and the compendium of target genes regulated through these co-receptors.

To understand how Wnt3a signaling regulates gene expression and to identify the roles of Lrp5 and Lrp6 in mediating Wnt3a signaling in osteoblasts, neonatal calvarial osteoblasts isolated from C57Bl6 (*WT*) and osteoblasts lacking either *Lrp5*, *Lrp6* or, both *Lrp5* and *6* were treated with Wnt3a for 24 hours (h) and gene expression changes were quantified by RNA sequencing (RNA-seq). We found that Wnt3a up-regulated the expression of several key regulators of osteoblast proliferation/early stages of differentiation while inhibiting genes highly expressed in later stages of osteoblastogenesis. We also found that Lrp6 is the key mediator of Wnt3a signaling in osteoblasts and loss of Lrp5 had minimal effect on Wnt3a signaling.

## Materials and methods

### Generation of knockout animals

All animal experimental procedures were completed in accordance with guidelines under the institutional animal care and use committees at Lawrence Livermore National Laboratory under an approved protocol by the IACUC committee, and conform to the NIH guide for the care and use of Laboratory animals. 3–5 days old pups from timed matings were genotyped by PCR as previously described [20]. *Lrp5* global knockout mice have been previously described and will be referred to herein as *Lrp5*^*KO*^ [21]. To generate *Lrp6* and dual *Lrp5/6* deficient osteoblasts, two previously described conditional alleles for *Lrp5* (*Lrp5*^*flox/flox*^) and *Lrp6* (*Lrp6*^*flox/flox*^) [11] were crossed to a tamoxifen inducible ubiquitous Cre recombinase transgenic strain of mice (UBC-Cre-ER^T2^; Jackson Laboratories, Bar Harbor, ME, USA) to generate *Lrp6*^*flox/flox*^*;UBC-Cre-ER*^*T2*^ and *Lrp5*^*flox/flox*^*;Lrp6*^*flox/flox*^*;UBC-Cre-ER*^*T2*^ mice; calvarial osteoblasts isolated from these pups (5–6 day old) and treated with hydroxytamoxifen (TMX), *in vitro*, will be referred to in this manuscript as *Lrp6*^*KO*^ OBs and *Lrp5/6*^*KO*^ OBs, respectively. *Lrp6*^*KO*^ OBs and *Lrp5/6*^*KO*^ OBs were compared to TMX treated *Lrp6*^*flox/flox*^ and *Lrp5*^*flox/flox*^*;Lrp6*^*flox/flox*^ OBs, respectively, which will be referred to in this manuscript as controls.

### Quantitative Real-time PCR

Total RNA was purified using RNeasy Mini Kit (QIAGEN Inc, Germantown, MD, USA) according to manufacturer’s protocol. Superscript III First-Strand Synthesis System (Invitrogen, Waltham, MA USA) was used with oligodT primers for reverse transcription according to manufacturer’s protocol. The qPCR was then performed with SYBR Select Master Mix (Applied Biosystems, Waltham, MA USA) using Applied Biosystems 7900HT Fast Real-Time PCR System with the following cycling conditions: 50°C for 2 min for SYBR then 95°C for 2 min, followed by 40 cycles of 95°C for 3 s and 30 s at 60°C. Data was normalized to control gene *Gapdh* and fold changes were calculated using the comparative Ct method [22]. Primers used for qPCR are given in S1 Table.

### Osteoblast isolation and culture

Osteoblasts were isolated from calvaria of neonates (5–6 days old) by serial digestion in Collagenase 1 as previously described [23]. Osteoblast enriched fractions were centrifuged, washed and plated in 12 well plates at 2.6X10^5 cells/well to ensure high cell number and subconfluency at the time of RNA collection. These cells were cultured in DMEM/F12 supplemented with 10% fetal bovine serum and 1% penicillin/streptomycin at 37°C. Following 24h incubation the media was changed to remove digestion debris and non-viable cells and these cultures were treated with 100 ng/ml recombinant Wnt3a (R&D systems, Minneapolis, MN, USA). The RNA was isolated from these cultures after incubating the cells for another 24h. A qPCR analysis was conducted on known Wnt target genes *Axin2*, *Lef1*, *Igfbp2* and *Ibh* and the Wnt3a activity was confirmed (S1 Fig). For *Lrp5/6*^*KO*^and *Lrp6*^*KO*^cells, following isolation of osteoblasts, cells were plated at 1x10^5 cells/well, cultured for 24h in DMEM+FBS+P/S media, followed by 48h treatment with DMEM+FBS+P/S with 1 uM hydroxytamoxifen (Sigma-Aldrich, St. Louis, MO, USA) prior to 24h Wnt3a treatment.

### RNA-isolation and sequencing

Total RNA was purified using RNeasy Mini Kit (QIAGEN Inc, Germantown, MD, USA) according to manufacturer’s protocol. RNA integrity was assessed using a bioanalyzer (Agilent Technologies, Santa Clara, CA, USA). Poly(A)+-enriched cDNA libraries were generated using the Illumina TruSeq Sample Preparation kit (Illumina Inc, Hayward, CA, USA) and checked for quality and quantity using the bioanalyzer and qPCR. The sequencing was performed using Illumina (Illumina Inc, Hayward, CA, USA) HiSeq 2000 (35-bp single-end reads) or NextSeq 550 (75-bp single-end reads). At least 3 replicates were generated for each experimental condition.

### RNA-seq data analysis

RNA sequence data quality was checked using FastQC (version 0.11.5) software. Trimmomatic (version 0. 30) [24] was used for data pre-processing. Using Trimmomatic, the reads were scanned with a 4-base wide sliding window, cutting when the average quality (Phread score) per base drops below 15. Reads with length <25 bases after preprocessing were discarded. Sequence reads were aligned to the mouse reference genome (mm10) using TopHat (version 2.0.11) [25, 26]. More than 90% of the reads were mapped to the mouse genome generating at least 15 million uniquely mapped reads/library. After read mapping, ‘featureCounts’ from Rsubread package (version 1.22.2) [27] was used to perform summarization of reads mapped to RefSeq genes and gene-wise read counts were generated. Genes were filtered from downstream analysis if they did not have a count per million mapped reads (CPM) value of at least 2 in at least five libraries. Subsequently, ‘TMM’ normalization was performed using the calcNormFactors function in edgeR (version 3.14.0) [28]. Differentially expressed genes were identified using ‘limma’ (version 3.28.12) after ‘voom’ [29] normalization. Experimental batch effects were adjusted for by including experimental batch as a covariate in the statistical model. A gene is called significantly differentially expressed when its false discovery rate adjusted p-value (FDR) is less than 0.05 and fold change is greater than 1.5.

BEDTools (version 2.26.0) [30] was used to count the number of RNA-seq reads mapped to deleted exons (exon2 in both *Lrp5* and *Lrp6*) in *Lrp6*^*KO*^ OBs, *Lrp5/6*^*KO*^ OBs and corresponding control samples. Cre-recombinase induced deletion was confirmed in all *Lrp6*^*KO*^ and *Lrp5/6*^*KO*^ RNA-seq samples by comparing the number of reads (in CPM) mapped to *Lrp5* exon2 and *Lrp6* exon2 in conditional knockout samples to corresponding control samples.

We also analyzed a publicly available RNA-seq dataset (GEO: GSE54461) profiling gene expression changes during differentiation of FACS sorted primary calvarial cells expressing Cyan Fluorescent protein (CFP) driven by a Col3.6 promoter to mature osteoblasts, and used this data to understand how the expression of Wnt3a targets change during differentiation of pre-osteoblasts to mature osteoblast. After aligning the RNA-seq reads to mouse genome (mm10) with TopHat [25, 26], gene expression values were calculated using Cuffnorm from Cufflinks (version 2.2.1) [31]. For Wnt3a targets, the expression values from all stages of differentiation were obtained from this dataset and clustered using hierarchical clustering to get clusters of genes with similar temporal expression profiles. Heatmaps were generated using heatmap.2 function in ‘gplots’ R package.

### Functional annotation

Gene ontology (GO) and pathway enrichment analysis was performed using functional annotation tool ToppGene [32] and enriched ‘biological processes’ and ‘pathways’ associated with differentially expressed genes were identified (FDR less than 0.05). ToppGene was also used to identify genes associated with bone phenotypes.

## Results

### Identification of Wnt3a-regulated transcriptome

**Fig 1:**
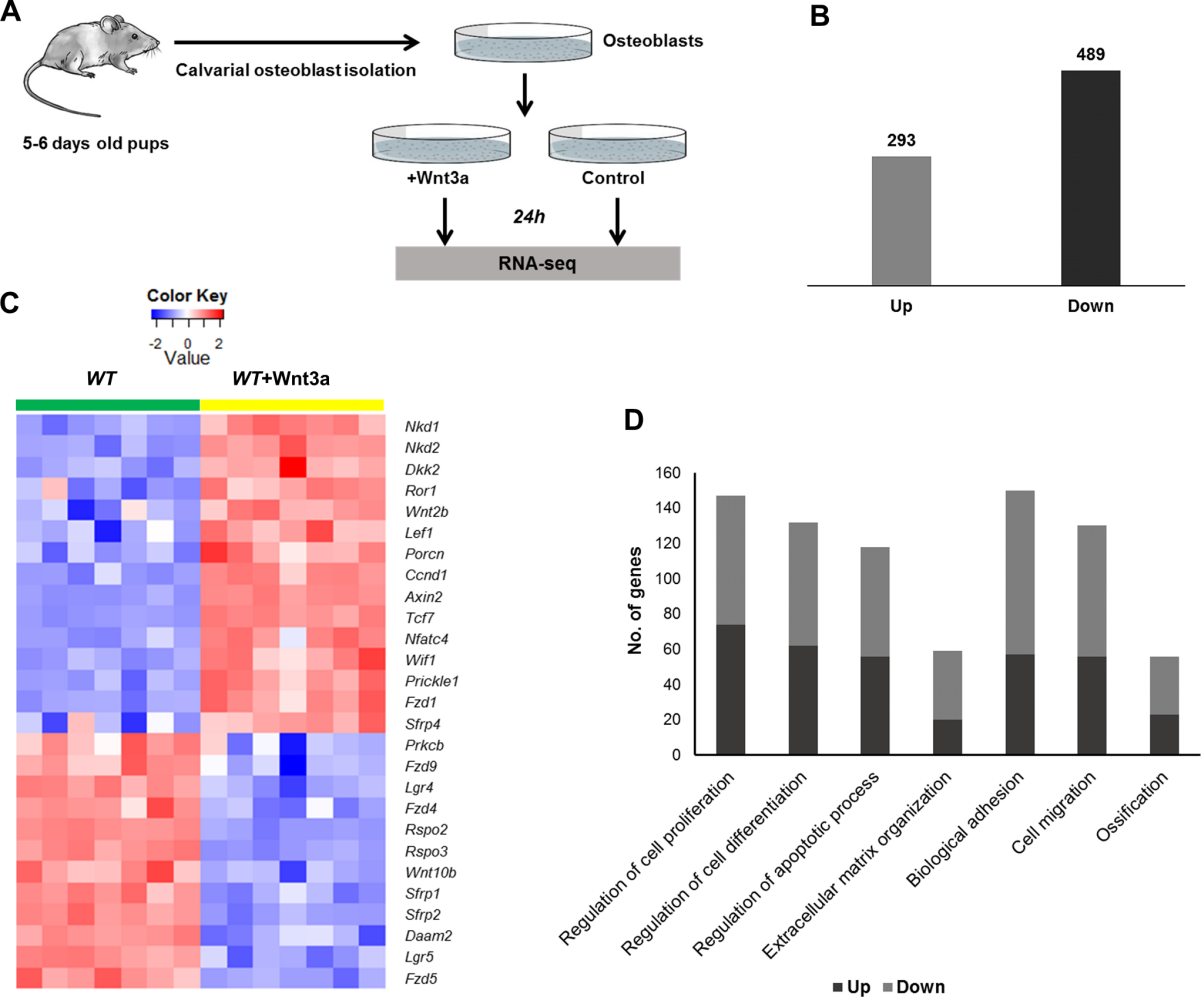
Identification of Wnt3a regulated transcriptome in osteoblasts. A) Experimental setup. Osteoblasts were isolated from calvaria of 5–6 days old mice and treated with Wnt3a recombinant protein. RNA was isolated from Wnt3a treated and sham treated cultures 24h post treatment, and sequenced using Illumina high-throughput sequencing technology. B) Genes up- and down-regulated by Wnt3a in osteoblasts. C) Heatmap shows the expression of differentially regulated Wnt pathway members in sham treated *WT* OBs (*WT*) and Wnt3a treated *WT* OBs (*WT*+Wnt3a). D) Enriched biological processes associated with up- and down-regulated genes. Figure shows the number of up- and down-regulated genes associated with relevant biological processes.

To investigate how Wnt3a regulates osteoblastic gene expression, neonatal calvarial osteoblasts from C57Bl/6 mice (*WT* OBs) were treated with 100 ng/ml recombinant Wnt3a for 24h and the gene expression changes were profiled using RNA-seq (Fig 1A). By comparing gene expression data from Wnt3a treated *WT* OBs to sham treated *WT* OBs we identified 293 up- and 489 down-regulated genes (Fig 1B, Table 1 and S2 Table). Several previously known canonical Wnt targets including *Axin2*, *Igfbp2*, *Cyr61*, *Lef1* and *Tnfrsf19* were identified as differentially expressed in osteoblasts in response to Wnt3a treatment. We also identified 33 growth factors, 15 transcription factors and 88 genes with receptor activity as Wnt3a targets in osteoblasts (S2 Table).

**Table 1:** Top 25 up- and down-regulated genes with highest log2 fold changes.

Up-regulated genes		Down-regulated genes	
*Gene*	*Fold change*	*Gene*	*Fold change*
*Bex1*	*4*.*388*	*Ucma*	*-4*.*600*
*Slc13a4*	*4*.*128*	*Stk32b*	*-3*.*975*
*Fgfbp1*	*3*.*782*	*Fmo1*	*-3*.*538*
*Sema4f*	*2*.*975*	*Angpt1*	*-3*.*035*
*Sstr2*	*2*.*844*	*Gprin3*	*-2*.*987*
*Nell2*	*2*.*826*	*Lect1*	*-2*.*863*
*Ndnf*	*2*.*799*	*Hgf*	*-2*.*763*
*Axin2*	*2*.*741*	*Scara5*	*-2*.*671*
*Ntf3*	*2*.*468*	*Sfrp2*	*-2*.*612*
*Tmem100*	*2*.*261*	*Rspo2*	*-2*.*491*
*Grik3*	*2*.*221*	*Matn4*	*-2*.*387*
*Igfbp2*	*2*.*187*	*Rspo3*	*-2*.*377*
*C430002N11Rik*	*2*.*139*	*Serpinb1a*	*-2*.*331*
*Aqp5*	*2*.*092*	*Entpd3*	*-2*.*230*
*Colec10*	*2*.*070*	*Cxcl12*	*-2*.*165*
*Syt13*	*2*.*059*	*Hp*	*-2*.*148*
*Tnfsf15*	*2*.*024*	*Dio3*	*-2*.*141*
*Hhip*	*2*.*012*	*Smoc1*	*-2*.*136*
*Dio2*	*1*.*971*	*Comp*	*-2*.*127*
*Nkd2*	*1*.*958*	*Palmd*	*-2*.*087*
*Slc1a2*	*1*.*926*	*Dner*	*-2*.*062*
*Bmper*	*1*.*925*	*Pparg*	*-2*.*059*
*Tgfb2*	*1*.*923*	*Chrdl1*	*-1*.*985*
*Grem1*	*1*.*888*	*Sox9*	*-1*.*932*
*Dpp4*	*1*.*883*	*Fzd9*	*-1*.*919*

Wnt3a altered the expression of 27 known genes from the Wnt signaling pathway: *Porcn*, *Fzd1*, *Lef1*, *Tcf7* and *Axin2* were up- while *Sfrp1*, *Sfrp2*, *Fzd4*, *Fzd9*, *Rspo2*, *Rspo3* and *Lgr4* were down-regulated in response to Wnt3a treatment (Fig 1C, Table 2). Wnt3a also regulated the expression of several members of TGF-β signaling pathway and MAPK signaling, two other key regulators of bone development and metabolism [33, 34] (Table 2, S3 Table).

**Table 2:** Key signaling pathways regulated by Wnt3a.

Signaling pathways	Genes regulated by Wnt3a
***Wnt signaling***	***Up*:** *Axin2*, *Fzd1*, *Tcf7*, *Sfrp4*, *Wnt2b*, *Nfatc4*, *Dkk2*, *Porcn*, *Ccnd1*, *Lef1*, *Prickle1*, *Wif1*, *Nkd1*, *Nkd2*, Ror1* Down: *Sfrp2*, *Fzd9*, *Fzd4*, *Fzd5*, *Sfrp1*, *Wnt10b*, *Prkcb*, *Daam2*, Rspo2*, Rspo3*, Lgr4*, Lgr5*
***TGF-β signaling***	***Up*:** *Tgfb2*, *Tgfb3*, *Bmp2*, *Bmp4*, *Bmp7*, *Inhba*, *Inhbb*, *Bmpr1b*, *Nog* ***Down*:** *Dcn*, *Id4*, *Fst*
***MAPK signaling***	***Up*:** *Cacna1c*, *Cacnb2*, *Dusp9*, *Fgfr1*, *Cacnb4*, *Cacna1g*, *Rras2*, *Dusp1*, *Fgf13*, *Fgf18*, *Tgfb3*, *Fgf21*, *Pdgfa*, *Ngf*, *Gadd45g*, *Tgfb2*, *Ntf3* ***Down***: *Cacna1d*, *Fgf7*, *Map2k6*, *Fgfr3*, *Pdgfra*, *Prkcb*, *Egfr*

*Genes obtained from http://web.stanford.edu/group/nusselab/cgi-bin/wnt/. Remaining genes were identified by functional annotation tool ToppGene.

GO analysis of differentially expressed genes showed enrichment for genes associated with several GO categories, including ‘regulation of cell proliferation’, ‘regulation of cell differentiation’, ‘extracellular matrix organization’, ‘biological adhesion’, ‘regulation of apoptotic process’, ‘cell migration’ and ‘ossification’ (Fig 1D, S4 Table). ‘Regulation of cell proliferation’ was one of the most significantly enriched terms for up-regulated genes with 74 genes including *Bmp2*, *Bmp4*, *Tgfb2*, *Pdgfa*, *Gdnf*, and *Hbegf* in that category (S4 Table). ‘Biological adhesion’ and ‘extracellular matrix organization’ were among the most significantly enriched GO terms associated with down-regulated genes (S4 Table). Wnt3a up-regulated 23 and down-regulated 33 genes associated with GO category ‘ossification’ (Table 3). We also identified several genes associated with bone phenotypes in mice as Wnt3a targets in osteoblasts. Forty-eight genes including *Bmp2*, *Fosl2* and *Cthrc1* with known bone phenotypes were up-regulated while 53 genes with bone phenotypes including *Spp1*, *Fzd9* and *Sfrp1* were down-regulated in response to Wnt3a treatment (Table 4).

**Table 3:** Wnt3a targets associated with GO term ‘ossification’.

Up-regulated genes			Down-regulated genes		
*Adrb2*	*Cthrc1*	*Lef1*	*Chrdl1*	*Gpm6b*	*Ostn*
*Axin2*	*Cyr61*	*Noct*	*Col11a2*	*Hgf*	*Penk*
*Bmp2*	*Enpp1*	*Nog*	*Col13a1*	*Id4*	*Ptn*
*Bmp3*	*Fgf18*	*Ptch1*	*Col2a1*	*Igf1*	*Rassf2*
*Tgfb2*	*Bmp4*	*Ptgs2*	*Dhrs3*	*Igsf10*	*Rspo2*
*Tgfb3*	*Bmp7*	*Grem1*	*Dlx5*	*Jag1*	*S1pr1*
*Rras2*	*Fgfr1*	*Bmpr1b*	*Sox9*	*Vcan*	*Egfr*
*Stc1*	*Fzd1*		*Spp1*	*Wnt10b*	*Fgfr3*
			*Srgn*	*Sfrp1*	*Gdf10*
			*Ucma*	*Sfrp2*	*Smoc1*
			*Kazald1*	*Lgr4*	*Mmp13*

**Table 4:** Wnt3a target genes associated with bone phenotypes in mice.

Up-regulated genes				Down-regulated genes			
*Enpp1*	*Dkk2*	*Errfi1*	*Tgfb2*	*Sfrp2*	*Meox2*	*Fam46a*	*Col2a1*
*Rictor*	*Cdo1*	*Ptch1*	*Dio2*	*Rspo2*	*Spp1*	*Id4*	*Hivep3*
*Sfrp4*	*Prrx2*	*Nov*	*Axin2*	*Rspo3*	*Cd74*	*Sfrp1*	*Mamld1*
*Rb1*	*Osbpl3*	*Ptgs2*	*Jak2*	*Comp*	*Dhrs3*	*Chrna7*	*Irak3*
*Itgb1*	*Sema3f*	*Slc20a1*	*Adrb2*	*Pparg*	*Spns2*	*Pdgfra*	*Ank1*
*Sgms1*	*Wbscr17*	*Lif*	*Prickle1*	*Sox9*	*Fgfr3*	*Vdr*	*Egfr*
*Bmp4*	*Bmp2*	*Hck*	*Fgf18*	*Fzd9*	*Itgb3*	*Rassf2*	*Tlr4*
*Cthrc1*	*Bmpr1b*	*Tcf7*	*Bmp7*	*Col14a1*	*Clstn3*	*Gnao1*	*Daam2*
*Pxylp1*	*Nog*	*Cnn1*	*Grem1*	*Penk*	*Dcn*	*Fmod*	*Xylt1*
*Mllt3*	*Galnt3*	*Grem2*	*Fosl2*	*C3*	*Dlx5*	*Ebf1*	*Fgf7*
*Fgfr1*	*Bmp3*	*Itga8*	*Smim3*	*Mmp13*	*Tlr2*	*Plagl1*	*Ednrb*
*Efemp1*	*Lepr*	*Tceal5*	*Col7a1*	*Cdkn1c*	*Epas1*	*Igf1*	
				*Col9a2*	*Pappa2*	*Epyc*	
				*Aldh3b1*	*Stat1*	*Npr3*	

### Wnt3a activates genes associated with early stages of osteogenesis and inhibits genes associated with late stage osteogenesis

**Fig 2:**
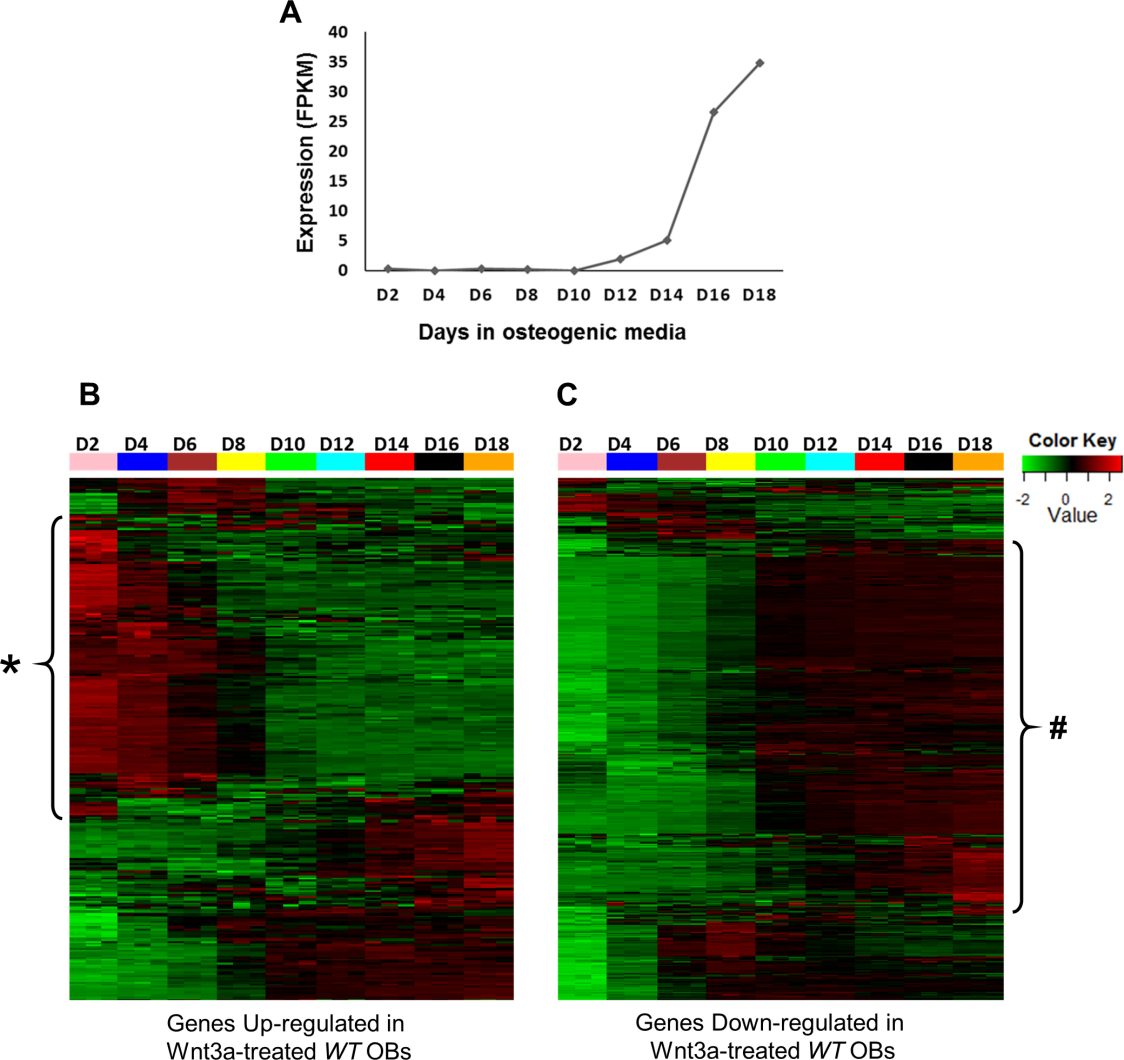
Expression profiles of Wnt3a targets during the differentiation of pre-osteoblast to mature osteoblasts. A) Expression profile of osteocalcin, a mature osteoblast marker. Osteocalcin expression starts around day 10. B) Expression profiles of genes up-regulated by Wnt3a. A large number of genes up-regulated by Wnt3a were highly expressed in early stage (D2-D8) osteoblasts (highlighted with *). C) Expression profiles of genes down-regulated by Wnt3a. Most of the genes down-regulated by Wnt3a were highly expressed in mature (D10-D18) osteoblasts (highlighted with #).

Temporal gene expression profiling across various stages of osteoblast differentiation can highlight cohort of transcripts with distinct roles during osteogenesis. To understand the temporal expression patterns of Wnt3a targets (identified above) during osteoblast differentiation, we analyzed the expression levels of these genes during the differentiation of purified pre-osteoblasts to mature osteoblasts capable of matrix mineralization (Fig 2A), by curating a publicly available dataset (GEO: GSE54461). This dataset includes RNA-seq from 2, 4, 6, 8, 10, 12, 14, 16 and 18 days post differentiation of pre-osteoblasts cultured in an osteoblast differentiation cocktail. Expression values of Wnt3a targets from all stages of osteoblast differentiation were obtained from this dataset and clustered using hierarchical clustering to get clusters of genes with similar temporal expression profiles. We determined that a large number (>62%) of genes up-regulated by Wnt3a were expressed at high levels during the early stages (2–8 days) of osteogenic differentiation (Fig 2B, S5 Table) whereas most of the genes down-regulated by Wnt3a were highly expressed in mature osteoblasts only (Fig 2C, S5 Table). Wnt3a also up-regulated several genes with higher expression in mature osteoblasts than immature osteoblasts including BMP/ TGF-β signaling pathway genes *Bmp2*, *Bmp3*, *Bmp4*, *Bmp7* and *Tgfb2*, FGF signaling pathway genes such as *Fgf13*, *Fgf18* and *Fgfr1* and, Wnt signaling pathway members including *Porcn*, *Axin2*, *Fzd1*, *Tcf7*, *Sfrp4*, *Nkd1* and *Prickle1* (Fig 2B, S5 Table). Several of these genes have been shown to regulate early stages of osteoblast differentiation as well as osteoblast maturation and mineralization [2, 35–38]. This data suggest that Wnt3a treatment promotes the expression of genes involved in osteoblast proliferation or early stages of differentiation while inhibiting genes involved in the later stages of osteoblastogenesis.

### Ablation of *Lrp5* and *Lrp6* has different effects on osteoblastic gene expression

**Fig 3:**
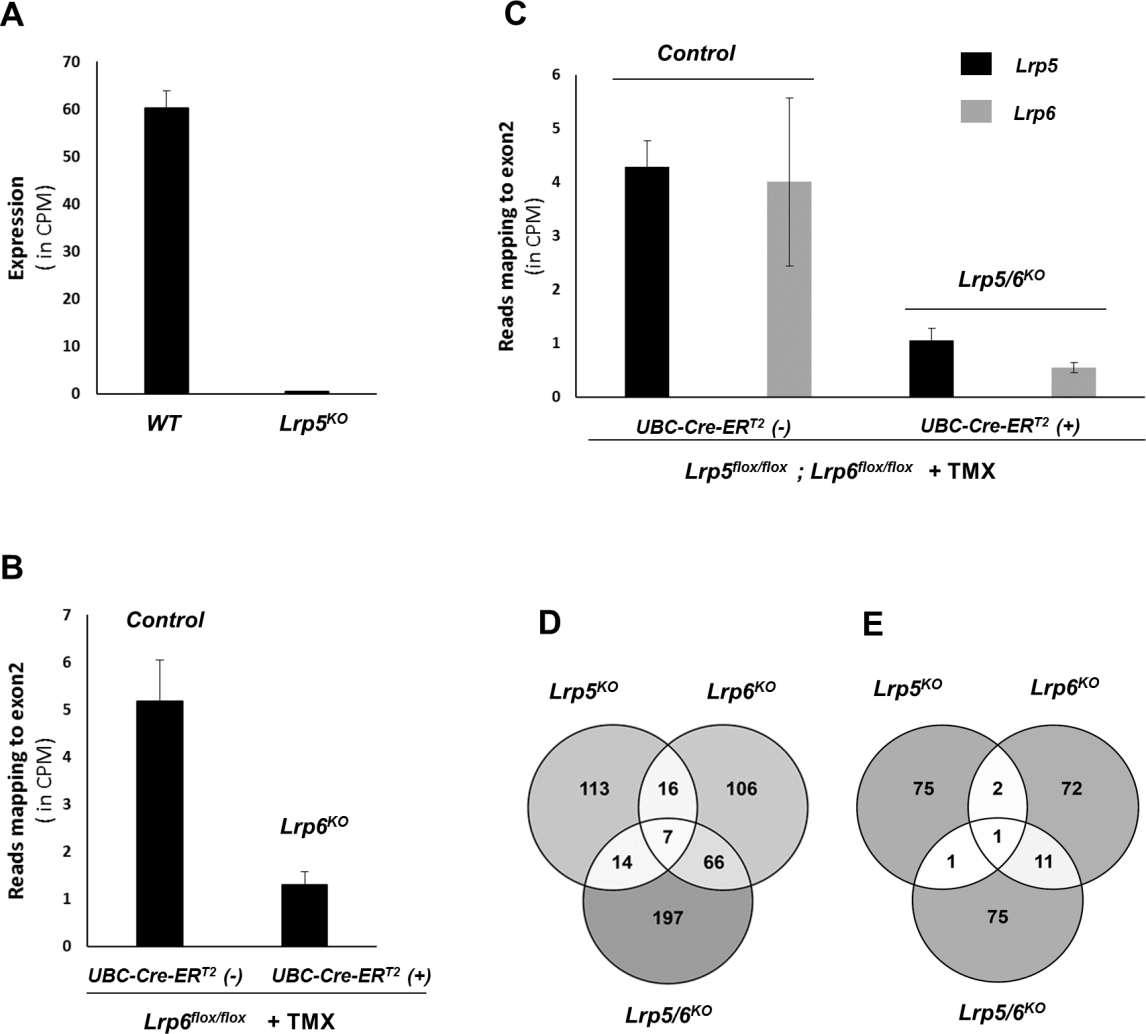
Effect of *Lrp5* and *Lrp6* ablation in osteoblasts. A) Expression of *Lrp5* in WT and *Lrp5*^*KO*^ OBs. Expression values are given in counts per million (CPM) mapped reads. B) Expression of *Lrp6* in controls (*Lrp6*^*fl/fl*^+TMX) and *Lrp6*^*KO*^ OBs (*Lrp6*^*fl/fl*^*;UBC-Cre-ER*^*T2*^+TMX). Expression values indicate the number of RNA-seq reads mapped to exon2 (deleted exon) in *Lrp6* (in CPM). C) Expression of *Lrp5* and *Lrp6* in controls (*Lrp5*^*fl/fl*^*;Lrp6*^*fl/fl*^+TMX) and *Lrp5/6*^*KO*^ OBs (*Lrp5*^*fl/fl*^*;Lrp6*^*fl/fl*^*; UBC-Cre-ER*^*T2*^+TMX). Expression values indicate the number of RNA-seq reads mapped to exon2 in *Lrp5* and *Lrp6* (in CPM). D) Venn diagram showing overlap between genes up-regulated in *Lrp5*^*KO*^ OBs, *Lrp6*^*KO*^ OBs and *Lrp5/6*^*KO*^ OBs compared to respective controls. E) Venn diagram showing overlap between genes down-regulated in *Lrp5*^*KO*^ OBs, *Lrp6*^*KO*^ OBs and *Lrp5/6*^*KO*^ OBs compared to respective controls.

To investigate the roles of Wnt co-receptors Lrp5 and Lrp6 in regulating gene expression in osteoblasts we generated *Lrp5* deficient (*Lrp5*^*KO*^), *Lrp6* deficient (*Lrp6*^*KO*^) and dual *Lrp5/6* deficient (*Lrp5/6*^*KO*^) osteoblasts. *Lrp5*^*KO*^ OBs were isolated from mice lacking *Lrp5* globally. *Lrp6*^*KO*^ OBs and *Lrp5/6*^*KO*^ OBs were generated *via in vitro* deletion of these genes in primary osteoblasts purified from floxed mice expressing *UBC-Cre-ER*^*T2*^ transgene. *Lrp5*^*KO*^ OB RNA-seq samples showed 99.3% reduction in *Lrp5* expression compared to *WT* OBs (Fig 3A). To account for the effects tamoxifen treatment (TMX) may have on gene expression, *Lrp6*^*KO*^and *Lrp5/6*^*KO*^ OBs were compared to TMX treated osteoblasts isolated from floxed littermates lacking the *UBC-Cre-ER*^*T2*^ transgene (controls). We found the expression of *Lrp6* to be reduced by ~75% in *Lrp6*^*KO*^ OB samples (Fig 3B), while *Lrp5* was ~76% and *Lrp6* was ~86% reduced in *Lrp5/6*^*KO*^ OBs (Fig 3C).

By comparing gene expression in *Lrp5*^*KO*^ OBs to *WT* OBs we identified 150 genes up- and 79 genes down-regulated in *Lrp5*^*KO*^ OBs (S6 Table). Next, we identified the top 50 enriched biological processes associated with differentially expressed genes. Thirty-nine genes associated with GO term ‘defense response’ including *Ccl7*, *Cxcl5*, *Tnfaip6* and *Ntrk2*, 28 genes associated with ‘cell migration’ including *Igfbp3*, *Ednrb*, *Dcn* and *Hgf*, 37 genes associated with ‘regulation of response to stress’ including *Xdh*, *Hgf*, *Casp4* and *Serpine2*, and 27 genes associated with ‘regulation of cell proliferation’ including *Cxcl12*, *Rbp4*, *Fabp4* and *Aldh3a1* were up-regulated in *Lrp5*^*KO*^ OBs (S7 Table). Down-regulated genes did not show enrichment for any biological processes. Key down-regulated genes include muscle genes *Actg2*, *Cnn1* and *Lmod1*, and *Pdpn*, a gene induced during osteoblast to osteocyte transition. Only 9 genes induced by Wnt3a in *WT* OB (identified above) including *Sfn*, *Ahr*, *Ankrd1*and *Ahrr* showed low expression in *Lrp5*^*KO*^ OBs relative to *WT* OBs while 25 genes down-regulated by Wnt3a in *WT* OBs including *Cxcl12*, *Dcn*, *Mmp13*, *Hgf* and *Mt2* showed increased expression in *Lrp5*^*KO*^ OBs (S6 Table).

By comparing *Lrp6*^*KO*^ to controls we identified 195 genes up- and 86 genes down-regulated in *Lrp6*^*KO*^(S6 Table). Genes up-regulated in *Lrp6*^*KO*^also showed enrichment for GO term ‘defense response’ with 41 genes including *Ntrk2*, *Il33* and *Mecom* in that category (S8 Table). Seventeen genes associated with ‘skeletal development’ including *Col2a1*, *Comp*, *Col9a1*and *Frzb* were also up-regulated in *Lrp6*^*KO*^. Other enriched GO categories include ‘cellular response to type I interferon (12 genes)’, ‘extracellular matrix organization (17 genes)’, ‘locomotion (34 genes)’, ‘apoptotic process (35)’ and ‘response to biotic stimulus (28 genes)’. Top 50 GO terms associated with down-regulated genes included ‘muscle cell differentiation (23 genes)’ and ‘actin cytoskeleton organization (14 genes)’. Five genes induced by Wnt3a in *WT* OB including *Grem1*, *Ndnf*, and *Dynap* were down-regulated and 70 genes down-regulated by Wnt3a in *WT* OBs including *Comp*, *Fzd9*, *Mmp13* and *F13a1* were up-regulated in *Lrp6*^*KO*^ OBs compared to controls (S6 Table).

Two hundred and eighty-four genes were up- and 88 genes were down-regulated in *Lrp5/6*^*KO*^ OBs compared to controls (S6 Table). Twenty-one genes induced by Wnt3a in *WT* OBs including *Axin2*, *Ndnf*, *Nkd1* and *Tnfrsf19* showed down-regulation in *Lrp5/6*^*KO*^ OBs while 84 genes down-regulated by Wnt3a in *WT* OBs were found to be up-regulated in *Lrp5/6*^*KO*^ OBs compared to controls (S6 Table).

**Fig 4:**
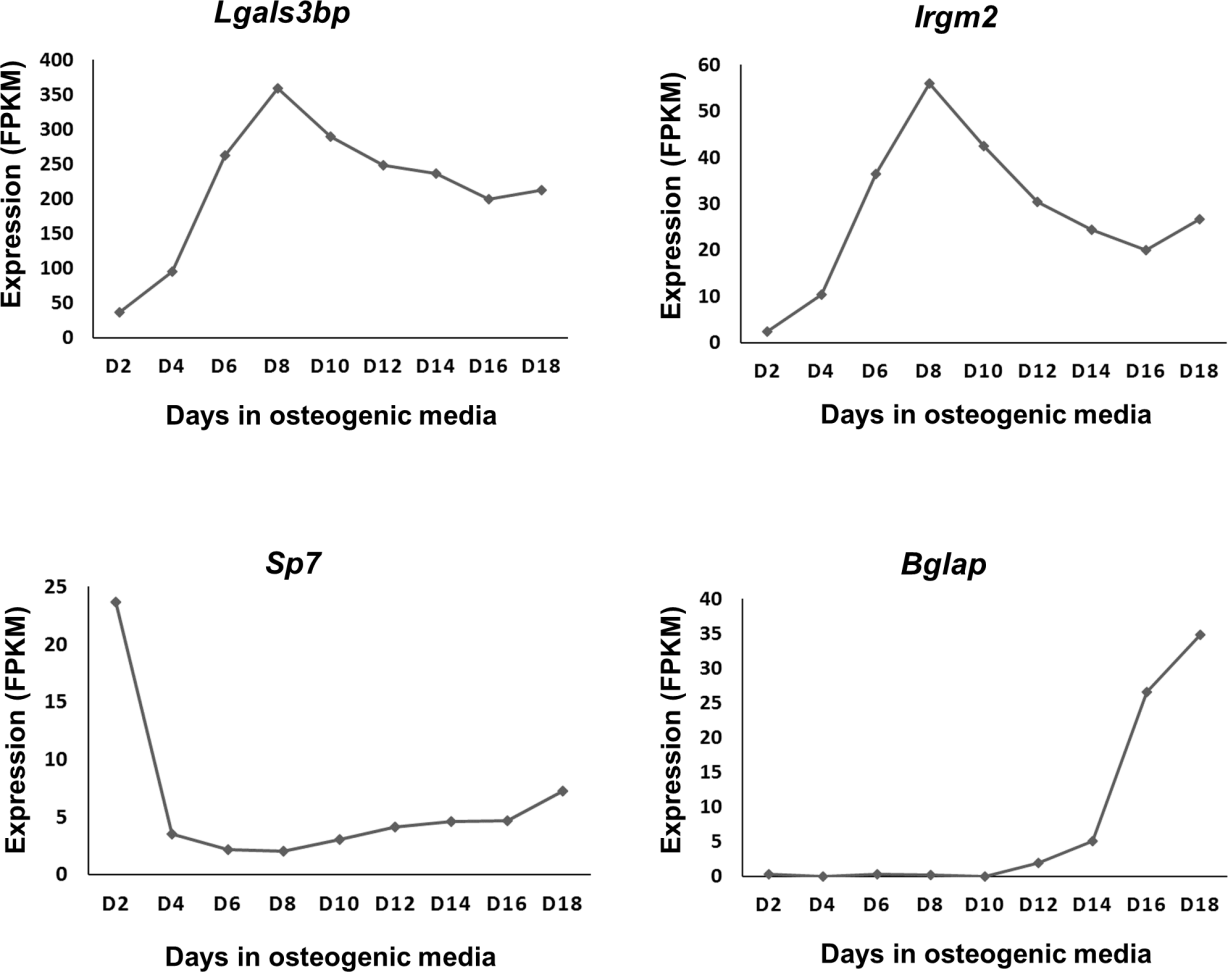
Expression profiles of *Lgals3bp* and *Irgm2* during osteoblast differentiation. Expression profiles of *Lgals3bp* and *Irgm2* compared to pre-osteoblast marker *Sp7* and mature osteoblast marker *Bglap* (Osteocalcin). Expression values were obtained from the RNA-seq dataset GSE54461. Both *Lgals3bp* and *Irgm2* were robustly expressed on osteoblasts and showed highest expression at day 8 (D8).

Of the 229 genes differentially expressed in *Lrp5*^*KO*^ OBs *vs*. *WT* OBs 26 (23 up; 3 down) and 23 (21 up; 2 down) genes overlapped with genes differentially expressed in *Lrp6*^*KO*^and *Lrp5/6*^*KO*^ OBs, respectively (Fig 3D and 3E, S6 Table). Eighty-five genes (73 up; 12 down) differentially expressed in *Lrp6*^*KO*^ *vs*. controls overlapped with genes differentially expressed in *Lrp5/6*^*KO*^ OBs relative to controls (Fig 3D and 3E, S6 Table). Eight genes (7 up; 1 down) including *Pou3f4*, *Ntrk2*, *Siglec1*, *Irgm2*, *Lgals3bp* (up) and *Col15a1* (down) were differentially expressed in all three receptor knockouts compared to respective controls (Fig 3D and 3E, S6 Table). Two of these up-regulated genes, *Irgm2* and *Lgals3bp* were also among the genes down-regulated by Wnt3a in *WT* OBs and, both genes exhibited very similar expression patterns during osteoblast differentiation with highest expression at day 8 (Fig 4).

### Ablation of *Lrp6* but, not *Lrp5*, significantly impaired Wnt3a signaling in osteoblasts

**Fig 5:**
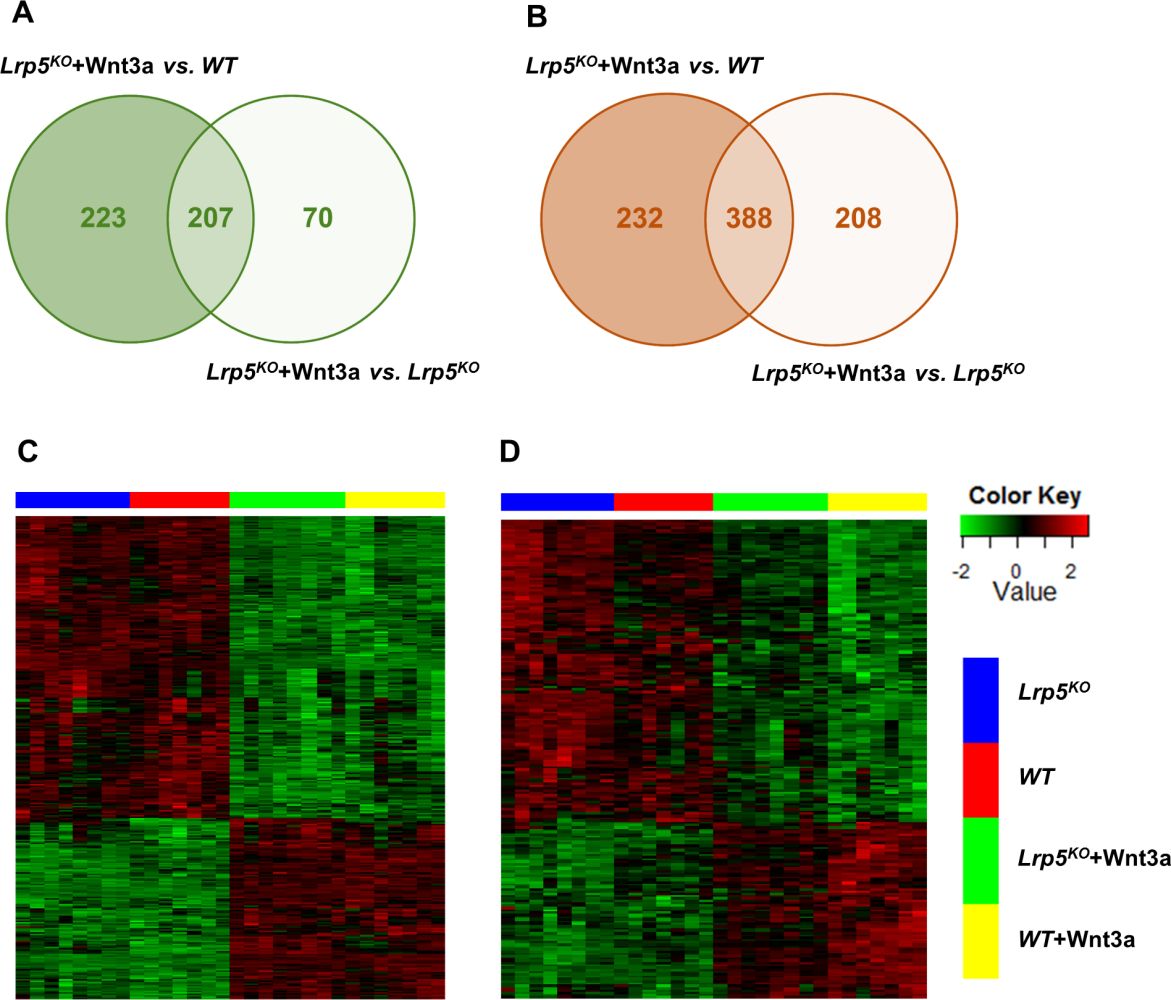
Wnt3a regulated genes in *Lrp5*^*KO*^ osteoblasts. A) Venn diagram showing overlap between genes up-regulated in Wnt3a treated *Lrp5*^*KO*^ OBs (*Lrp5*^*KO*^+Wnt3a) compared to sham treated *WT* OBs (*WT*) and sham treated *Lrp5*^*KO*^ OBs (*Lrp5*^*KO*^). B) Venn diagram showing overlap between genes down-regulated in Wnt3a treated *Lrp5*^*KO*^ OBs (*Lrp5*^*KO*^+Wnt3a) compared to sham treated *WT* OBs (*WT*) and sham treated *Lrp5*^*KO*^ OBs (*Lrp5*^*KO*^). C) Heatmap showing genes up- or down-regulated by Wnt3a in both *WT* OBs (*WT* +Wnt3a) and *Lrp5*^*KO*^ OBs (*Lrp5*^*KO*^+Wnt3a) compared to *WT* (*WT*) controls. D) Heatmap showing genes up- or down-regulated by Wnt3a in *WT* OBs (*WT* +Wnt3a) but, not in *Lrp5*^*KO*^ OBs (*Lrp5*^*KO*^+Wnt3a) compared to *WT* (*WT*) controls. These genes either had a fold change < 1.5 or FDR corrected p-value > 0.05.

To investigate the role of Lrp5 and Lrp6 in mediating Wnt3a signaling in osteoblasts we quantified the gene expression changes in Wnt3a treated *Lrp5*^*KO*^ (*Lrp5*^*KO*^*+*Wnt3a), *Lrp6*^*KO*^(*Lrp6*^*KO*^*+*Wnt3a) and *Lrp5/6*^*KO*^(*Lrp5/6*^*KO*^*+*Wnt3a) OBs compared to respective sham treated controls. We identified 1050 (430 up; 620 down) differentially expressed genes in *Lrp5*^*KO*^*+*Wnt3a compared to sham treated *WT* OBs (*Lrp5*^*KO*^+Wnt3a *vs*. *WT* OBs; S9 Table). This included genes regulated by Wnt3a independent of Lrp5 and genes that may not be Wnt3a dependent but, changed as a result of loss of Lrp5. Four hundred and fifty-five out of the 1050 genes differentially expressed in *Lrp5*^*KO*^+Wnt3a *vs*. *WT* OBs were not significantly differentially expressed in *Lrp5*^*KO*^+Wnt3a *vs*. *Lrp5*^*KO*^ OBs (Fig 5A and 5B), suggesting that their expression is Lrp5 dependent, and Wnt3a treatment may not have a significant impact on the expression of some of these genes. Only 88 of these 455 genes were identified as Wnt3a targets in *WT* OBs (identified above as differentially expressed in *WT* OBs +Wnt3a *vs*. *WT* OBs), suggesting that the remaining genes may not be Wnt3a dependent. It is possible that these genes are regulated by other Wnt ligands such as Wnt1 and Wnt10b *via* Lrp5 or a complex interplay between Wnts and other signaling pathways and, loss of Lrp5 resulted in up- or down-regulation of these genes.

**Fig 6:**
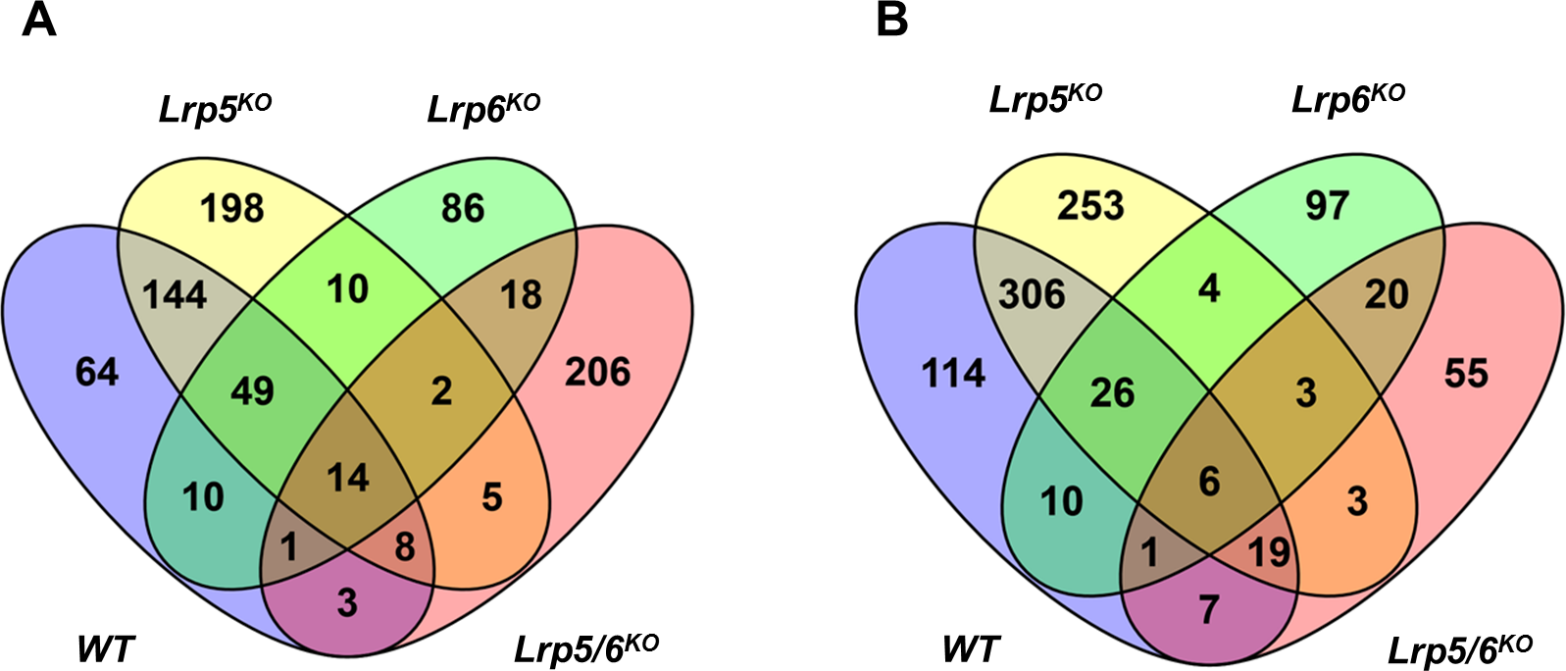
Overlap between Wnt3a targets identified in *WT*, *Lrp5*^*KO*^, *Lrp6*^*KO*^ and *Lrp5/6*^*KO*^ osteoblasts. A) Overlap between genes up-regulated by Wnt3a. B) Overlap between genes down-regulated by Wnt3a.

About 73% (572/782) of the Wnt3a targets identified in *WT* OBs (identified above as differentially expressed in *WT* OBs +Wnt3a *vs*. *WT* OBs) were also significantly differentially expressed in *Lrp5*^*KO*^ OBs in response to Wnt3a treatment (*Lrp5*^*KO*^+Wnt3a *vs*. *WT OBs*), suggesting that Wnt3a signaling is minimally affected by the loss of *Lrp5* in osteoblasts (Figs 5C & 6, S9 Table). Majority of the remaining Wnt3a targets also showed up- or down-regulation in *Lrp5*^*KO*^ +Wnt3a *vs*. *WT* OBs although the changes were below the significance threshold used in this study (Fig 5D), suggesting that Lrp5 is required for the optimal Wnt3a-mediated activation or repression of these genes.

By comparing *Lrp6*^*KO*^*+*Wnt3a to sham treated controls, we identified 357 (190 up, 167 down) differentially expressed genes (S9 Table). Two hundred and fifty-five of these genes were not differentially expressed in *Lrp6*^*KO*^*+*Wnt3a *vs*. *Lrp6*^*KO*^suggesting that their expression is Lrp6 dependent and may not directly depend on Wnt3a. Only ~15% (117/782) of the Wnt3a targets identified in *WT* OBs (identified above as differentially expressed in *WT* OBs +Wnt3a *vs*. *WT* OBs) were found to be differentially expressed between Wnt3a treated *Lrp6*^*KO*^ OBs and sham treated controls, suggesting that the lack of *Lrp6* significantly impaired Wnt3a signaling in osteoblasts (Fig 6, S9 Table).

Three hundred and seventy-one (257 up; 114 down) genes were differentially expressed between *Lrp5/6*^*KO*^*+*Wnt3a and sham treated controls (S9 Table). However, only ~8% (59/782) of the Wnt3a target genes including *Inhbb*, *Sema4f* and *Nell2* were found to be significantly differentially expressed in Wnt3a treated *Lrp5/6*^*KO*^ OBs compared to sham treated controls (Fig 6, S9 Table). This suggests that Wnt3a-dependent signaling is greatly impaired in osteoblasts lacking both *Lrp5* and *Lrp6*.

## Discussion

Several studies have shown that Wnt3a plays a key role in skeletal development and bone metabolism [8, 39]. Although numerous Wnt3a target genes have been identified in different cell types including pluripotent mesenchymal cell line C3H10T1/2 [40] and stromal cell line ST2 [41], very little is known about the target genes regulated by Wnt3a in primary osteoblasts. This study provides the first account of Wnt3a regulated transcriptome in primary osteoblasts.

We identified 782 Wnt3a targets in osteoblasts including 101 genes with bone phenotypes in mice, highlighting the importance of Wnt3a signaling in regulating skeletal development and bone metabolism. Previous studies have shown that Wnt signaling induces the expression of the osteoblast marker alkaline phosphatase (*Alp*) [42]. Although *Alp* and other osteoblast markers such as *Sp7* and *Runx2* were expressed in the primary murine osteoblast used in this study, we did not observe any significant changes in the expression of these genes in response to treatment with Wnt3a for 24 hours. However, culturing these Wnt3a stimulated cells for a longer period or treating these cells with recombinant Wnt3a for a longer duration might lead to an elevated expression of *Alp* and other osteoblast markers in these cells.

Our study identified several members of Wnt signaling pathway as Wnt3a targets, suggesting a feedback regulatory mechanism mediated by Wnt3a in osteoblasts. Wnt3a also regulated the expression of members of other signaling pathways including BMP/ TGF-β signaling and MAPK signaling, several growth factors including *Pdgfa*, *Hbegf*, *Ngf* and *Ntf3*, and transcription factors including *Hdac9*, *Ankrd1*, *Vdr*, *Pparg*, *Sox9* and *Nfatc4*. It is reasonable to speculate that Wnt3a indirectly regulated the expression of some of the genes identified in this study through the activation of other pathways and transcription factors. The expression of *Pparg*, a key regulator of adipogenesis, was >4-fold down in Wnt3a treated osteoblasts. Reduced *Pparg* expression is associated with increased bone formation and, canonical Wnt signaling has been shown to down-regulate *Pparg* [41, 43]. Previous studies have also shown that loss of canonical Wnt signaling causes cell fate shift of pre-osteoblasts from osteoblasts to adipocytes [44]. Our data suggest that Wnt3a may repress the differentiation of pre-osteoblasts to adipocytes by suppressing *Pparg* expression. Wnt3a also down-regulated *Sox9*, a transcription factor that regulates chondrocyte differentiation [45]. In mice, overexpression of *Sox9* in osteoblasts from a 2.3-kb *Col1a1* promoter resulted in dwarfism and osteopenia, with a significant reduction in bone volume, osteoblasts number and bone formation rate [45]. Bone marrow stromal cells isolated from *Sox9* transgenic mice also displayed enhanced adipocyte differentiation and decreased osteoblast differentiation *in vitro* [45]. This data suggest that *Sox9* is a negative regulator of osteoblast differentiation and bone formation. Down-regulation of *Sox9* in osteoblasts by Wnt3a may also contribute to enhanced osteogenesis and bone formation.

Few *in vitro* studies have suggested that Wnt3a promotes osteoblast differentiation [41, 46, 47]. In contrast, several studies have reported that Wnt3a promotes osteoblast proliferation and suppresses osteoblasts differentiation [8, 48]. Examination of the temporal expression patterns of Wnt3a targets during osteoblastogenesis revealed that a large number (>62%) of genes up-regulated by Wnt3a are generally highly expressed during the early stages of osteogenic differentiation and the majority of the genes down-regulated by Wnt3a are highly expressed in mature osteoblasts. This data suggests that Wnt3a promotes osteoblast proliferation or early stages of osteoblast differentiation and inhibits osteoblast maturation/mineralization. This is in line with previous findings by Boland *et al*. [8] and Caverzasio *et al*. [48] that Wnt3a enhances proliferation of MSCs, pre-osteoblast cell lines and mouse primary osteoblasts [8]. Boland *et al*. have also shown that Wnt3a treatment inhibited mineralization of MSCs that had been osteogenically differentiated for 12 days prior to Wnt3a exposure and this effect was reversible [8]. Our study identified several regulators of early stage osteogenesis induced by Wnt3a including *Pdgfa* [49], *Cyr61* [50] and *Tgfb3* [51] and regulators of osteoblast maturation/mineralization repressed by Wnt3a including *Vdr* [52] and *Rspo2* [53]. Wnt3a also up-regulated a number of genes with higher expression in mature osteoblasts than early stage osteoblasts including several members of BMP/TGF-β signaling pathway (*Bmp2*, *Bmp3*, *Bmp7*, *Tgfb2* etc.), FGF signaling pathway (*Fgf13*, *Fgf18*, *Fgfr1* etc.) and Wnt signaling pathway (*Porcn*, *Axin2*, *Fzd1*, *Tcf7*, *Sfrp4*, *Nkd1*, *Prickle1* etc.). Many of these genes have been shown to regulate both early and late stages of osteoblast differentiation [2, 35–38]. This suggests that some of the Wnt3a activated genes identified in this study may play a role in both early and late stages of osteogenesis.

Lrp5 and Lrp6 are key mediators of β-catenin dependent Wnt signaling. However, very little is known about their specific roles in regulating gene expression in osteoblasts. We found very little overlap between genes differentially regulated in the absence of *Lrp5* and *Lrp6* in osteoblasts suggesting that these two receptors have non-redundant functions in regulating osteoblastic gene expression. We found that the expression of several known canonical Wnt targets were not dramatically altered in osteoblasts lacking either *Lrp5* or *Lrp6*; however, osteoblasts lacking both *Lrp5* and *Lrp6* showed down-regulation of several known canonical Wnt targets including *Axin2*, *Nkd1* and *Tnfrsf19*. Two genes up-regulated in sham treated *Lrp5*^*KO*^ OBs, *Lrp6*^*KO*^ OBs and *Lrp5/6*^*KO*^ OBs compared to respective sham treated controls, *Irgm2* and *Lgals3bp*, were also found to be down-regulated by Wnt3a in *WT* OBs and, both genes had very similar expression profiles during osteoblast differentiation with highest expression at day 8 (Fig 4). *Irgm2* is a GTPase and *Lgals3bp* is involved in cell–cell and cell–matrix interactions. However, their functions in bone is not known. It is likely that these genes play a significant role in regulating osteoblastogenesis.

We also found that a large number of genes suppressed by Wnt3a in *WT* OBs were up-regulated in sham treated *Lrp5*^*KO*^, *Lrp6*^*KO*^ and *Lrp5/6*^*KO*^ OBs compared to respective sham treated controls. However, only few genes up-regulated by Wnt3a were down-regulated in osteoblasts lacking these receptors. The total number of genes suppressed by Wnt3a in *WT* OBs were also significantly higher than the number of Wnt3a activated genes (489 down *vs*. 293 up). This suggests that transcriptional repression is a major mechanism by which Wnt3a preforms its functions in osteoblasts. This is consistent with a recent report by Karner *et al*. that, in stromal cell line ST2, Wnt3a activated significantly fewer genes compared to the number of genes it inhibited [41]. They also showed that Wnt3a inhibited gene expression by suppressing histone acetylation possibly in an Lrp5/6 dependent but, β-catenin independent manner. Further studies are required to understand the exact mechanism by which canonical Wnt signaling suppress gene expression in osteoblasts, and how these outcomes are interconnected with other molecular pathways.

Our study also showed that that Wnt3a regulated >73% of its target genes independent of Lrp5 whereas lack of Lrp6 significantly impaired the ability of Wnt3a to regulate target gene expression. This suggests that Lrp6 is the key mediator of Wnt3a signaling in osteoblasts. However, global genetic deletion of *Lrp5* greatly impairs bone metabolism, suggesting that *Lrp5* deficiency does play a significant role in bone. Our data suggests that Lrp5 may mediate its signaling *via* other Wnt ligands, and such candidates include Wnt1 and Wnt10b; alternatively, Lrp5 may have a context dependent function in bone. ~8% (59/782) of the Wnt3a target genes including *Inhbb*, *Sema4f* and *Nell2* were also differentially expressed in Wnt3a treated *Lrp5/6*^*KO*^ OBs compared to sham treated controls. It is likely that these genes are activated *via* non-canonical Wnt pathways as Wnt3a has previously been shown to activate non-canonical pathways [54].

The analysis of gene expression changes is a powerful approach for elucidating the molecular mechanisms by which signaling pathways regulate biological processes such as bone metabolism. However, the current study is limited in its examination of the role of Wnt3a signaling in neonatal calvarial osteoblasts 24h post treatment. Future studies could include osteoblasts isolated from adult mice, osteoblasts from different skeletal locations and osteoblasts from various stages of differentiation treated with Wnts for varying duration to get a more detailed picture of canonical Wnt signaling in osteoblasts. Also, a large number of Wnt targets identified in this study have not been previously characterized in the context of bone metabolism. Further studies are required to determine their impact on osteoblast function and bone metabolism. Overall, the data presented herein will further our understanding of the role of the canonical Wnt signaling pathway in the regulation of osteoblast differentiation and function and in addition, this study will enhance current knowledge of the Wnt signaling pathway itself.

## Supporting information

S1 Fig Confirming Wnt3a activity. A qPCR analysis showed that Wnt3a up-regulated known canonical Wnt target genes *Axin2*, *Lef1*, *Igfbp2* and down-regulated *Ibh*, a gene suppressed by canonical Wnt signaling.
(TIF)

S1 Table qPCR primers used in this study. (PDF)

S2 Table Genes up- or down-regulated by Wnt3a. Fold changes are given in log2 scale.
(XLSX)

S3 Table Key signaling pathways associated with Wnt3a targets. (PDF)

S4 Table Enriched GO terms associated with Wnt3a targets. (PDF)

S5 Table Expression of Wnt3a targets during the differentiation of pre-osteoblasts to mature osteoblasts. (XLSX)

S6 Table Genes differentially-regulated in Lrp5/Lrp6 receptor knockouts compared to respective controls. Fold changes are given in log2 scale. Empty cells represent no significant change in expression.
(XLSX)

S7 Table Top 50 enriched GO terms associated with genes differentially expressed in *Lrp5*^*KO*^ osteoblasts compared to *WT* osteoblasts. (PDF)

S8 Table Top 50 enriched GO terms associated with genes differentially expressed in *Lrp6*^*KO*^ osteoblasts compared to *Lrp6*^*fl/fl*^ + TMX. (PDF)

S9 Table Genes differentially-regulated in Lrp5/Lrp6 receptor knockouts treated with Wnt3a compared to respective controls. Fold changes are given in log2 scale. Empty cells represent no significant change in expression.
(XLSX)
