# Supplementary figures and images for: Wnt co-receptors Lrp5 and Lrp6 differentially mediate Wnt3a signaling in osteoblasts
Source: PLoS One. 2017 Nov 27;12(11):e0188264. doi: 10.1371/journal.pone.0188264 (PMC5703471; doi:10.1371/journal.pone.0188264)

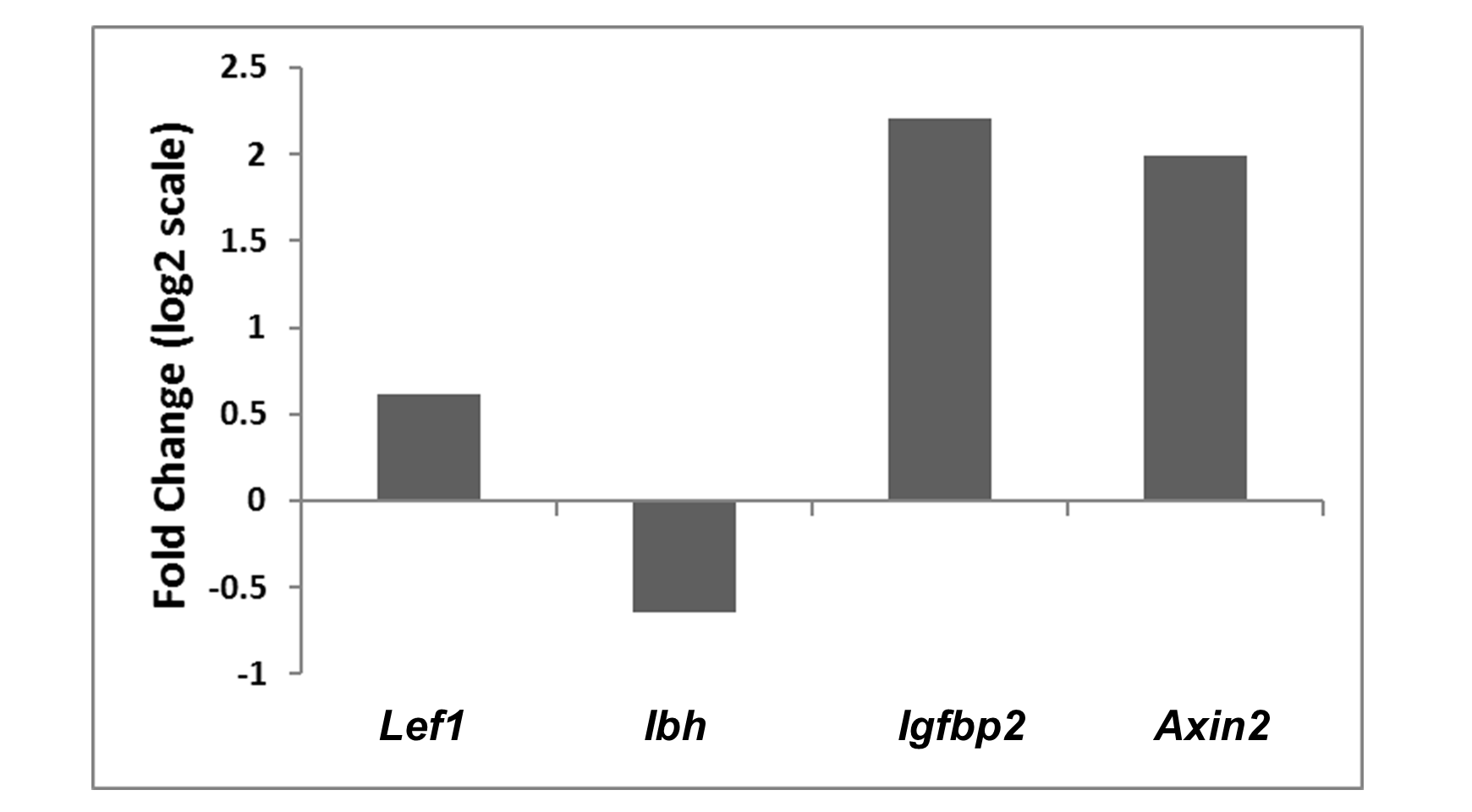

Supplement: S1 Fig — A qPCR analysis showed that Wnt3a up-regulated known canonical Wnt target genes Axin2, Lef1, Igfbp2 and down-regulated Ibh, a gene suppressed by canonical Wnt signaling. (TIF) [file pone.0188264.s001.tif]
