# Supplementary material for: Wnt co-receptors Lrp5 and Lrp6 differentially mediate Wnt3a signaling in osteoblasts
Source: PLoS One. 2017 Nov 27;12(11):e0188264. doi: 10.1371/journal.pone.0188264 (PMC5703471; doi:10.1371/journal.pone.0188264)
Supplement: S1 Table — (PDF) [file pone.0188264.s002.pdf]

S1 Table. qPCR primers used in this study

	Forward	***Reverse***
Axin2	*ATGAGTAGCGCCGTGTTAGTG*	*GGGCATAGGTTTGGTGGACT*
Igfbp2	*CAGACGCTACGCTGCTATCC*	*CTCCCTCAGAGTGGTCGTCA*
Lef1	*ATCACCTACAGCGACGAGCAC*	*TGGACATGGAAGTGTCGCCTG*
Ibh	*CTGCTCTGACTATCTGAGATCGG*	*CAGCAACGGTCAAAGTCTGAT*
Gapdh	CCAATGTGTCCGTCGTGGATCT	CCTCAGTGTAGCCCAAGATGC
