# Supplementary material for: Wnt co-receptors Lrp5 and Lrp6 differentially mediate Wnt3a signaling in osteoblasts
Source: PLoS One. 2017 Nov 27;12(11):e0188264. doi: 10.1371/journal.pone.0188264 (PMC5703471; doi:10.1371/journal.pone.0188264)
Supplement: S3 Table — (PDF) [file pone.0188264.s004.pdf]

S3 Table. Key signaling pathways associated with Wnt3a targets

Enriched pathways associated with up-regulated genes		
Pathway	***No. of genes***	***FDR***
MAPK signaling pathway	*17*	*1.66E-04*
Hippo signaling pathway	*15*	*9.43E-06*
Cytokine-cytokine receptor interaction	*15*	*2.15E-03*
Wnt signaling pathway	*14*	*1.98E-05*
TGF-beta signaling pathway	*9*	*7.47E-04*
p53 signaling pathway	*6*	*2.80E-02*
integrin signaling	*5*	*1.47E-02*
Signaling by BMP	*4*	*2.03E-02*

Enriched pathways associated with down-regulated genes		
Pathway	***No. of genes***	***FDR***
Cytokine Signaling in Immune system	*34*	*4.44E-02*
PI3K-Akt signaling pathway	*24*	*8.39E-04*
Rap1 signaling pathway	*15*	*1.63E-02*
Integrin signalling pathway	*13*	*1.71E-02*
Toll-like receptor signaling pathway	*10*	*1.61E-02*
Interferon gamma signaling	*9*	*2.67E-02*
EphrinA-EPHA pathway	*4*	*2.17E-03*
