# Supplementary material for: Wnt co-receptors Lrp5 and Lrp6 differentially mediate Wnt3a signaling in osteoblasts
Source: PLoS One. 2017 Nov 27;12(11):e0188264. doi: 10.1371/journal.pone.0188264 (PMC5703471; doi:10.1371/journal.pone.0188264)
Supplement: S4 Table — (PDF) [file pone.0188264.s005.pdf]

## S4 Table. Enriched GO terms associated with Wnt3a targets

| GO terms associted with up-regulated genes | | | |
|---|---|---|---|
| ***GO ID*** | ***Enriched Biological Process*** | ***No. genes*** | ***FDR*** |
| *GO:0042127* | *regulation of cell proliferation* | *74* | *8.32E-16* |
| *GO:0048729* | *tissue morphogenesis* | *48* | *4.25E-15* |
| *GO:0048646* | *anatomical structure formation involved in morphogenesis* | *62* | *1.81E-14* |
| *GO:0001944* | *vasculature development* | *44* | *2.48E-14* |
| *GO:0007167* | *enzyme linked receptor protein signaling pathway* | *53* | *9.46E-14* |
| *GO:0051094* | *positive regulation of developmental process* | *61* | *1.03E-13* |
| *GO:0001525* | *angiogenesis* | *35* | *3.59E-13* |
| *GO:0009719* | *response to endogenous stimulus* | *70* | *3.59E-13* |
| *GO:0060485* | *mesenchyme development* | *27* | *4.59E-13* |
| *GO:0023057* | *negative regulation of signaling* | *58* | *2.73E-12* |
| *GO:0048585* | *negative regulation of response to stimulus* | *63* | *2.73E-12* |
| *GO:0051270* | *regulation of cellular component movement* | *46* | *2.75E-12* |
| *GO:0048762* | *mesenchymal cell differentiation* | *23* | *3.58E-12* |
| *GO:0040007* | *growth* | *52* | *3.58E-12* |
| *GO:0010648* | *negative regulation of cell communication* | *58* | *3.58E-12* |
| *GO:0030334* | *regulation of cell migration* | *42* | *5.47E-12* |
| *GO:0007267* | *cell-cell signaling* | *55* | *7.67E-12* |
| *GO:0048863* | *stem cell differentiation* | *27* | *8.20E-12* |
| *GO:2000145* | *regulation of cell motility* | *43* | *9.86E-12* |
| *GO:0040012* | *regulation of locomotion* | *45* | *1.01E-11* |
| *GO:0016477* | *cell migration* | *56* | *1.50E-11* |
| *GO:0009968* | *negative regulation of signal transduction* | *53* | *1.52E-11* |
| *GO:0090130* | *tissue migration* | *24* | *1.95E-11* |
| *GO:0071363* | *cellular response to growth factor stimulus* | *38* | *1.95E-11* |
| *GO:0009967* | *positive regulation of signal transduction* | *60* | *2.51E-11* |
| *GO:0070848* | *response to growth factor* | *38* | *6.73E-11* |
| *GO:0006928* | *movement of cell or subcellular component* | *68* | *7.07E-11* |
| *GO:0045597* | *positive regulation of cell differentiation* | *46* | *8.75E-11* |
| *GO:0051674* | *localization of cell* | *57* | *1.42E-10* |
| *GO:0048870* | *cell motility* | *57* | *1.42E-10* |
| *GO:0051093* | *negative regulation of developmental process* | *44* | *1.78E-10* |
| *GO:0000904* | *cell morphogenesis involved in differentiation* | *42* | *1.78E-10* |
| *GO:0008284* | *positive regulation of cell proliferation* | *44* | *2.45E-10* |
| *GO:0014031* | *mesenchymal cell development* | *20* | *3.04E-10* |
| *GO:0008285* | *negative regulation of cell proliferation* | *38* | *3.33E-10* |
| *GO:0040011* | *locomotion* | *63* | *3.88E-10* |
| *GO:0045595* | *regulation of cell differentiation* | *62* | *4.71E-10* |
| *GO:0048864* | *stem cell development* | *20* | *4.92E-10* |
| *GO:0023056* | *positive regulation of signaling* | *61* | *5.54E-10* |
| *GO:0010647* | *positive regulation of cell communication* | *61* | *9.31E-10* |
| *GO:0030335* | *positive regulation of cell migration* | *29* | *1.07E-09* |
| *GO:0040017* | *positive regulation of locomotion* | *30* | *1.11E-09* |

| | | | |
|---|---|---|---|
| GO:0010941 | regulation of cell death | 60 | 1.15E-09 |
| GO:2000147 | positive regulation of cell motility | 29 | 1.99E-09 |
| GO:0022610 | biological adhesion | 57 | 2.12E-09 |
| GO:0007178 | transmembrane receptor protein serine/threonine kinase signaling pathway | 25 | 2.46E-09 |
| GO:0051272 | positive regulation of cellular component movement | 29 | 3.41E-09 |
| GO:0042981 | regulation of apoptotic process | 56 | 3.41E-09 |
| GO:0060393 | regulation of pathway-restricted SMAD protein phosphorylation | 12 | 4.01E-09 |
| GO:0007155 | cell adhesion | 56 | 4.38E-09 |
| GO:0043067 | regulation of programmed cell death | 56 | 4.97E-09 |
| GO:0012501 | programmed cell death | 65 | 4.97E-09 |
| GO:0001932 | regulation of protein phosphorylation | 53 | 4.97E-09 |
| GO:0060389 | pathway-restricted SMAD protein phosphorylation | 12 | 6.73E-09 |
| GO:0006915 | apoptotic process | 64 | 7.15E-09 |
| GO:0042325 | regulation of phosphorylation | 55 | 7.40E-09 |
| GO:0001763 | morphogenesis of a branching structure | 20 | 1.56E-08 |
| GO:0040008 | regulation of growth | 35 | 1.86E-08 |
| GO:0014070 | response to organic cyclic compound | 43 | 2.61E-08 |
| GO:0090092 | regulation of transmembrane receptor protein serine/threonine kinase signaling pathway | 19 | 3.08E-08 |
| GO:0006468 | protein phosphorylation | 63 | 3.08E-08 |
| GO:0002040 | sprouting angiogenesis | 12 | 3.25E-08 |
| GO:0030178 | negative regulation of Wnt signaling pathway | 18 | 4.64E-08 |
| GO:0090287 | regulation of cellular response to growth factor stimulus | 20 | 4.94E-08 |
| GO:0009725 | response to hormone | 42 | 5.31E-08 |
| GO:0010720 | positive regulation of cell development | 30 | 5.31E-08 |
| GO:0051130 | positive regulation of cellular component organization | 47 | 8.21E-08 |
| GO:0030111 | regulation of Wnt signaling pathway | 22 | 8.37E-08 |
| GO:0007169 | transmembrane receptor protein tyrosine kinase signaling pathway | 33 | 1.01E-07 |
| GO:0031399 | regulation of protein modification process | 59 | 1.34E-07 |
| GO:0007389 | pattern specification process | 27 | 1.47E-07 |
| GO:0051247 | positive regulation of protein metabolic process | 54 | 1.53E-07 |
| GO:0030324 | lung development | 18 | 1.53E-07 |
| GO:0019220 | regulation of phosphate metabolic process | 57 | 1.61E-07 |
| GO:0032989 | cellular component morphogenesis | 50 | 1.61E-07 |
| GO:0051216 | cartilage development | 17 | 1.69E-07 |
| GO:0042327 | positive regulation of phosphorylation | 40 | 1.96E-07 |
| GO:0023014 | signal transduction by protein phosphorylation | 39 | 1.96E-07 |
| GO:0030029 | actin filament-based process | 32 | 1.96E-07 |
| GO:0051174 | regulation of phosphorus metabolic process | 57 | 1.96E-07 |
| GO:0043065 | positive regulation of apoptotic process | 31 | 2.04E-07 |
| GO:0043068 | positive regulation of programmed cell death | 31 | 2.47E-07 |
| GO:0031401 | positive regulation of protein modification process | 45 | 2.55E-07 |
| GO:0010942 | positive regulation of cell death | 32 | 2.63E-07 |
| GO:0048705 | skeletal system morphogenesis | 18 | 2.67E-07 |

| | | | |
|---|---|---|---|
| GO:0030278 | regulation of ossification | 17 | 2.94E-07 |
| GO:1904035 | regulation of epithelial cell apoptotic process | 11 | 3.01E-07 |
| GO:0060284 | regulation of cell development | 39 | 3.43E-07 |
| GO:0000902 | cell morphogenesis | 47 | 3.72E-07 |
| GO:0060688 | regulation of morphogenesis of a branching structure | 10 | 3.82E-07 |
| GO:0061448 | connective tissue development | 19 | 4.02E-07 |
| GO:0045765 | regulation of angiogenesis | 18 | 4.30E-07 |
| GO:0071772 | response to BMP | 15 | 5.37E-07 |
| GO:0071773 | cellular response to BMP stimulus | 15 | 5.37E-07 |
| GO:1902531 | regulation of intracellular signal transduction | 56 | 5.72E-07 |
| GO:0072132 | mesenchyme morphogenesis | 9 | 6.57E-07 |
| GO:0006935 | chemotaxis | 28 | 6.83E-07 |
| GO:0042330 | taxis | 28 | 7.31E-07 |
| GO:0003002 | regionalization | 22 | 7.41E-07 |
| GO:0032270 | positive regulation of cellular protein metabolic process | 50 | 7.77E-07 |
| GO:0001503 | ossification | 23 | 7.77E-07 |
| GO:0061061 | muscle structure development | 30 | 8.47E-07 |
| GO:0010769 | regulation of cell morphogenesis involved in differentiation | 22 | 8.75E-07 |
| GO:0030855 | epithelial cell differentiation | 30 | 9.90E-07 |
| GO:0061035 | regulation of cartilage development | 10 | 1.06E-06 |
| GO:0030155 | regulation of cell adhesion | 31 | 1.09E-06 |
| GO:0048608 | reproductive structure development | 25 | 1.18E-06 |
| GO:0010562 | positive regulation of phosphorus metabolic process | 41 | 1.25E-06 |
| GO:0045937 | positive regulation of phosphate metabolic process | 41 | 1.25E-06 |
| GO:0061458 | reproductive system development | 25 | 1.35E-06 |
| GO:0071407 | cellular response to organic cyclic compound | 27 | 1.44E-06 |
| GO:0016049 | cell growth | 27 | 1.48E-06 |
| GO:0043627 | response to estrogen | 17 | 1.62E-06 |
| GO:0001501 | skeletal system development | 26 | 1.73E-06 |
| GO:0003156 | regulation of animal organ formation | 8 | 2.04E-06 |
| GO:0045926 | negative regulation of growth | 18 | 2.25E-06 |
| GO:0033993 | response to lipid | 38 | 2.37E-06 |
| GO:0048545 | response to steroid hormone | 27 | 2.43E-06 |
| GO:0090090 | negative regulation of canonical Wnt signaling pathway | 14 | 3.48E-06 |
| GO:0030036 | actin cytoskeleton organization | 27 | 3.78E-06 |
| GO:0010594 | regulation of endothelial cell migration | 12 | 4.17E-06 |
| GO:0072089 | stem cell proliferation | 14 | 4.23E-06 |
| GO:0048589 | developmental growth | 29 | 4.30E-06 |
| GO:0060537 | muscle tissue development | 22 | 4.58E-06 |
| GO:0072078 | nephron tubule morphogenesis | 10 | 5.21E-06 |
| GO:0045667 | regulation of osteoblast differentiation | 12 | 5.76E-06 |
| GO:0030198 | extracellular matrix organization | 20 | 6.05E-06 |
| GO:0031589 | cell-substrate adhesion | 19 | 6.25E-06 |
| GO:0043062 | extracellular structure organization | 20 | 6.26E-06 |
| GO:0048666 | neuron development | 39 | 6.50E-06 |
| GO:0051384 | response to glucocorticoid | 14 | 7.51E-06 |
| GO:0045859 | regulation of protein kinase activity | 31 | 7.51E-06 |

| | | | |
|---|---|---|---|
| GO:0032355 | response to estradiol | 13 | 8.08E-06 |
| GO:0090100 | positive regulation of transmembrane receptor protein serine/threonine kinase signaling pathway | 11 | 8.57E-06 |

| GO terms associted with down-regulated genes | | | |
|---|---|---|---|
| GO ID | Enriched Biological Process | No. genes | FDR |
| GO:0022610 | biological adhesion | 93 | 1.44E-14 |
| GO:0007155 | cell adhesion | 89 | 4.36E-13 |
| GO:0030198 | extracellular matrix organization | 39 | 6.86E-13 |
| GO:0043062 | extracellular structure organization | 39 | 6.86E-13 |
| GO:0048514 | blood vessel morphogenesis | 48 | 2.09E-12 |
| GO:0001525 | angiogenesis | 42 | 3.05E-11 |
| GO:1903034 | regulation of response to wounding | 42 | 4.66E-11 |
| GO:0001568 | blood vessel development | 50 | 4.66E-11 |
| GO:0001944 | vasculature development | 51 | 4.85E-11 |
| GO:0040011 | locomotion | 90 | 5.57E-11 |
| GO:0032101 | regulation of response to external stimulus | 64 | 7.75E-11 |
| GO:0051241 | negative regulation of multicellular organismal process | 68 | 9.4E-11 |
| GO:0016477 | cell migration | 74 | 1.15E-10 |
| GO:0006952 | defense response | 86 | 1.15E-10 |
| GO:0080134 | regulation of response to stress | 83 | 1.87E-10 |
| GO:0051674 | localization of cell | 77 | 3.87E-10 |
| GO:0048870 | cell motility | 77 | 3.87E-10 |
| GO:0030334 | regulation of cell migration | 51 | 7.26E-10 |
| GO:0009611 | response to wounding | 59 | 1.67E-09 |
| GO:0006928 | movement of cell or subcellular component | 90 | 2.47E-09 |
| GO:0048585 | negative regulation of response to stimulus | 78 | 2.49E-09 |
| GO:2000145 | regulation of cell motility | 51 | 6.98E-09 |
| GO:0031347 | regulation of defense response | 52 | 6.98E-09 |
| GO:0002682 | regulation of immune system process | 76 | 9.78E-09 |
| GO:0040012 | regulation of locomotion | 53 | 1.49E-08 |
| GO:0045087 | innate immune response | 52 | 1.9E-08 |
| GO:0043207 | response to external biotic stimulus | 57 | 2.19E-08 |
| GO:0051270 | regulation of cellular component movement | 52 | 3.1E-08 |
| GO:0009607 | response to biotic stimulus | 58 | 3.43E-08 |
| GO:0002237 | response to molecule of bacterial origin | 31 | 4.29E-08 |
| GO:0001503 | ossification | 33 | 4.97E-08 |
| GO:0006955 | immune response | 76 | 5.41E-08 |
| GO:0009967 | positive regulation of signal transduction | 73 | 6.6E-08 |
| GO:0010647 | positive regulation of cell communication | 79 | 8.59E-08 |
| GO:0023056 | positive regulation of signaling | 78 | 1.08E-07 |
| GO:0048589 | developmental growth | 43 | 2E-07 |
| GO:0006935 | chemotaxis | 39 | 2.24E-07 |
| GO:0030155 | regulation of cell adhesion | 44 | 2.36E-07 |

| | | | |
|---|---|---|---|
| GO:0042330 | taxis | 39 | 2.36E-07 |
| GO:0051240 | positive regulation of multicellular organismal process | 75 | 3.03E-07 |
| GO:0006954 | inflammatory response | 44 | 3.03E-07 |
| GO:0009617 | response to bacterium | 39 | 4.13E-07 |
| GO:0001816 | cytokine production | 43 | 5.56E-07 |
| GO:0040007 | growth | 57 | 5.92E-07 |
| GO:0050727 | regulation of inflammatory response | 28 | 7.12E-07 |
| GO:1902533 | positive regulation of intracellular signal transduction | 52 | 8.16E-07 |
| GO:0032722 | positive regulation of chemokine production | 12 | 8.31E-07 |
| GO:0032103 | positive regulation of response to external stimulus | 27 | 1.2E-06 |
| GO:0051093 | negative regulation of developmental process | 50 | 1.2E-06 |
| GO:0030335 | positive regulation of cell migration | 32 | 1.31E-06 |
| GO:0002684 | positive regulation of immune system process | 52 | 1.31E-06 |
| GO:0009968 | negative regulation of signal transduction | 59 | 1.31E-06 |
| GO:0040017 | positive regulation of locomotion | 33 | 1.82E-06 |
| GO:0001817 | regulation of cytokine production | 39 | 1.98E-06 |
| GO:0001501 | skeletal system development | 35 | 2.09E-06 |
| GO:2000147 | positive regulation of cell motility | 32 | 2.27E-06 |
| GO:0032496 | response to lipopolysaccharide | 27 | 2.35E-06 |
| GO:0031589 | cell-substrate adhesion | 26 | 2.67E-06 |
| GO:0050678 | regulation of epithelial cell proliferation | 26 | 2.8E-06 |
| GO:0032642 | regulation of chemokine production | 13 | 3.51E-06 |
| GO:0002687 | positive regulation of leukocyte migration | 16 | 3.58E-06 |
| GO:0051272 | positive regulation of cellular component movement | 32 | 3.69E-06 |
| GO:0001818 | negative regulation of cytokine production | 22 | 3.74E-06 |
| GO:0045088 | regulation of innate immune response | 28 | 3.74E-06 |
| GO:0042127 | regulation of cell proliferation | 73 | 3.93E-06 |
| GO:0031349 | positive regulation of defense response | 30 | 4.57E-06 |
| GO:0033993 | response to lipid | 52 | 4.57E-06 |
| GO:0045824 | negative regulation of innate immune response | 10 | 4.94E-06 |
| GO:1901700 | response to oxygen-containing compound | 71 | 4.94E-06 |
| GO:1903036 | positive regulation of response to wounding | 19 | 4.94E-06 |
| GO:0070374 | positive regulation of ERK1 and ERK2 cascade | 19 | 5.78E-06 |
| GO:0045596 | negative regulation of cell differentiation | 40 | 6.42E-06 |
| GO:0023057 | negative regulation of signaling | 61 | 6.66E-06 |
| GO:0032602 | chemokine production | 13 | 6.78E-06 |
| GO:0050776 | regulation of immune response | 47 | 8.32E-06 |
| GO:0071346 | cellular response to interferon-gamma | 16 | 9.16E-06 |
| GO:0002526 | acute inflammatory response | 17 | 9.84E-06 |
| GO:0010648 | negative regulation of cell communication | 61 | 9.86E-06 |
| GO:0048646 | anatomical structure formation involved in morphogenesis | 60 | 1.03E-05 |
| GO:0060349 | bone morphogenesis | 13 | 1.07E-05 |
| GO:0050920 | regulation of chemotaxis | 19 | 1.07E-05 |
| GO:0010721 | negative regulation of cell development | 26 | 1.24E-05 |
| GO:0044236 | multicellular organism metabolic process | 16 | 1.26E-05 |
| GO:0034341 | response to interferon-gamma | 17 | 1.31E-05 |
| GO:0044259 | multicellular organismal macromolecule metabolic process | 15 | 1.32E-05 |

| | | | |
|---|---|---|---|
| GO:0048608 | reproductive structure development | 31 | 1.65E-05 |
| GO:0030278 | regulation of ossification | 19 | 1.71E-05 |
| GO:0050900 | leukocyte migration | 27 | 1.79E-05 |
| GO:0061458 | reproductive system development | 31 | 1.89E-05 |
| GO:0050729 | positive regulation of inflammatory response | 15 | 2.07E-05 |
| GO:0048729 | tissue morphogenesis | 41 | 2.22E-05 |
| GO:0002697 | regulation of immune effector process | 30 | 2.25E-05 |
| GO:0048699 | generation of neurons | 66 | 2.29E-05 |
| GO:0002252 | immune effector process | 44 | 2.37E-05 |
| GO:0043410 | positive regulation of MAPK cascade | 32 | 2.65E-05 |
| GO:0001763 | morphogenesis of a branching structure | 20 | 3.21E-05 |
| GO:0002685 | regulation of leukocyte migration | 17 | 3.42E-05 |
| GO:0046903 | secretion | 56 | 3.45E-05 |
| GO:0008284 | positive regulation of cell proliferation | 46 | 3.53E-05 |
| GO:0045765 | regulation of angiogenesis | 20 | 3.54E-05 |
| GO:0050730 | regulation of peptidyl-tyrosine phosphorylation | 20 | 3.74E-05 |
| GO:0032963 | collagen metabolic process | 14 | 3.94E-05 |
| GO:1901342 | regulation of vasculature development | 21 | 4.11E-05 |
| GO:0044093 | positive regulation of molecular function | 76 | 4.21E-05 |
| GO:0060828 | regulation of canonical Wnt signaling pathway | 20 | 4.57E-05 |
| GO:0001649 | osteoblast differentiation | 19 | 4.73E-05 |
| GO:0045595 | regulation of cell differentiation | 70 | 4.78E-05 |
| GO:0010810 | regulation of cell-substrate adhesion | 17 | 5.65E-05 |
| GO:0071345 | cellular response to cytokine stimulus | 38 | 6.26E-05 |
| GO:0033002 | muscle cell proliferation | 17 | 0.000069 |
| GO:0002683 | negative regulation of immune system process | 27 | 7.24E-05 |
| GO:0030111 | regulation of Wnt signaling pathway | 23 | 7.38E-05 |
| GO:0050921 | positive regulation of chemotaxis | 14 | 7.67E-05 |
| GO:0001101 | response to acid chemical | 25 | 7.78E-05 |
| GO:0045785 | positive regulation of cell adhesion | 27 | 8.59E-05 |
| GO:0023014 | signal transduction by protein phosphorylation | 46 | 8.73E-05 |
| GO:0048659 | smooth muscle cell proliferation | 14 | 9.56E-05 |
| GO:0032940 | secretion by cell | 50 | 9.8E-05 |
| GO:0070371 | ERK1 and ERK2 cascade | 21 | 0.000112 |
| GO:1902531 | regulation of intracellular signal transduction | 71 | 0.000113 |
| GO:0043408 | regulation of MAPK cascade | 38 | 0.000114 |
| GO:0050679 | positive regulation of epithelial cell proliferation | 16 | 0.000115 |
| GO:0010941 | regulation of cell death | 67 | 0.000119 |
| GO:0051094 | positive regulation of developmental process | 57 | 0.000123 |
| GO:0018108 | peptidyl-tyrosine phosphorylation | 25 | 0.000129 |
| GO:0050731 | positive regulation of peptidyl-tyrosine phosphorylation | 16 | 0.00013 |
| GO:0071396 | cellular response to lipid | 32 | 0.000137 |
| GO:0018212 | peptidyl-tyrosine modification | 25 | 0.000139 |
| GO:0060350 | endochondral bone morphogenesis | 9 | 0.000151 |
| GO:2000027 | regulation of organ morphogenesis | 20 | 0.000154 |
| GO:0032490 | detection of molecule of bacterial origin | 5 | 0.000157 |
| GO:0070372 | regulation of ERK1 and ERK2 cascade | 20 | 0.000161 |

| | | | |
|---|---|---|---|
| GO:0001934 | positive regulation of protein phosphorylation | 45 | 0.000163 |
| GO:0048771 | tissue remodeling | 16 | 0.000163 |
| GO:0002009 | morphogenesis of an epithelium | 33 | 0.000178 |
| GO:0031214 | biomineral tissue development | 14 | 0.000179 |
| GO:0032680 | regulation of tumor necrosis factor production | 13 | 0.000179 |
| GO:0045667 | regulation of osteoblast differentiation | 13 | 0.000179 |
| GO:0030199 | collagen fibril organization | 8 | 0.00018 |
| GO:0003006 | developmental process involved in reproduction | 37 | 0.000198 |
| GO:0050778 | positive regulation of immune response | 35 | 0.000203 |
| GO:0042327 | positive regulation of phosphorylation | 46 | 0.000215 |
| GO:0042981 | regulation of apoptotic process | 62 | 0.000215 |
| GO:1903555 | regulation of tumor necrosis factor superfamily cytokine production | 13 | 0.000223 |
| GO:0048705 | skeletal system morphogenesis | 18 | 0.00023 |
| GO:0044243 | multicellular organismal catabolic process | 10 | 0.000233 |
| GO:0034097 | response to cytokine | 40 | 0.00024 |
| GO:0050918 | positive chemotaxis | 9 | 0.000241 |
| GO:0030282 | bone mineralization | 12 | 0.00025 |
| GO:0098609 | cell-cell adhesion | 45 | 0.000252 |
| GO:0042592 | homeostatic process | 69 | 0.000259 |
| GO:0071295 | cellular response to vitamin | 7 | 0.000259 |
| GO:1900046 | regulation of hemostasis | 11 | 0.000261 |
| GO:0045089 | positive regulation of innate immune response | 21 | 0.000264 |
| GO:0048660 | regulation of smooth muscle cell proliferation | 13 | 0.000268 |
| GO:0071706 | tumor necrosis factor superfamily cytokine production | 13 | 0.000268 |
| GO:0032757 | positive regulation of interleukin-8 production | 8 | 0.000269 |
| GO:0043067 | regulation of programmed cell death | 62 | 0.000278 |
| GO:0014066 | regulation of phosphatidylinositol 3-kinase signaling | 14 | 0.000292 |
| GO:0003416 | endochondral bone growth | 6 | 0.000308 |
| GO:0060348 | bone development | 16 | 0.000311 |
| GO:0042325 | regulation of phosphorylation | 61 | 0.000315 |
| GO:0051336 | regulation of hydrolase activity | 57 | 0.000325 |
| GO:0050663 | cytokine secretion | 16 | 0.000325 |
| GO:0032677 | regulation of interleukin-8 production | 9 | 0.000329 |
| GO:0006915 | apoptotic process | 73 | 0.00033 |
| GO:0010628 | positive regulation of gene expression | 72 | 0.000337 |
| GO:0042116 | macrophage activation | 9 | 0.000363 |
| GO:0010562 | positive regulation of phosphorus metabolic process | 49 | 0.000363 |
| GO:0045937 | positive regulation of phosphate metabolic process | 49 | 0.000363 |
| GO:0043085 | positive regulation of catalytic activity | 63 | 0.000368 |
| GO:1901701 | cellular response to oxygen-containing compound | 45 | 0.000373 |
| GO:0006024 | glycosaminoglycan biosynthetic process | 12 | 0.000373 |
