# Supplementary material for: Wnt co-receptors Lrp5 and Lrp6 differentially mediate Wnt3a signaling in osteoblasts
Source: PLoS One. 2017 Nov 27;12(11):e0188264. doi: 10.1371/journal.pone.0188264 (PMC5703471; doi:10.1371/journal.pone.0188264)
Supplement: S7 Table — (PDF) [file pone.0188264.s008.pdf]

S7 Table. Top 50 enriched GO terms associated with genes differentially expressed in *Lrp5* ^{KO} osteoblasts compared to *WT* osteoblasts

Genes up-regulated in *Lrp5* ^{KO} Osteoblasts			
GO ID	***Enriched Biological Process***	***No. genes***	***FDR***
GO:0006952	*defense response*	*39*	*1.16E-11*
GO:0080134	*regulation of response to stress*	*37*	*4.97E-11*
GO:0002684	*positive regulation of immune system process*	*29*	*1.25E-10*
GO:0006954	*inflammatory response*	*25*	*1.61E-10*
GO:0031347	*regulation of defense response*	*25*	*2.99E-09*
GO:0050727	*regulation of inflammatory response*	*17*	*5.48E-09*
GO:0032101	*regulation of response to external stimulus*	*27*	*5.79E-09*
GO:1903034	*regulation of response to wounding*	*19*	*9.18E-09*
GO:0032103	*positive regulation of response to external stimulus*	*16*	*2.16E-08*
GO:0051270	*regulation of cellular component movement*	*24*	*2.69E-08*
GO:0006955	*immune response*	*32*	*3.42E-08*
GO:0030334	*regulation of cell migration*	*22*	*4.70E-08*
GO:0002682	*regulation of immune system process*	*31*	*4.70E-08*
GO:0030335	*positive regulation of cell migration*	*17*	*1.34E-07*
GO:2000145	*regulation of cell motility*	*22*	*1.34E-07*
GO:0016477	*cell migration*	*28*	*1.34E-07*
GO:2000147	*positive regulation of cell motility*	*17*	*1.88E-07*
GO:0051674	*localization of cell*	*29*	*2.01E-07*
GO:0048870	*cell motility*	*29*	*2.01E-07*
GO:0051272	*positive regulation of cellular component movement*	*17*	*2.36E-07*
GO:0040017	*positive regulation of locomotion*	*17*	*2.81E-07*
GO:0031349	*positive regulation of defense response*	*16*	*4.14E-07*
GO:0040012	*regulation of locomotion*	*22*	*4.68E-07*
GO:0002687	*positive regulation of leukocyte migration*	*10*	*4.76E-07*
GO:0050729	*positive regulation of inflammatory response*	*10*	*5.71E-07*
GO:0009611	*response to wounding*	*23*	*5.98E-07*
GO:0002685	*regulation of leukocyte migration*	*11*	*6.52E-07*
GO:0050900	*leukocyte migration*	*15*	*6.52E-07*
GO:1903036	*positive regulation of response to wounding*	*11*	*1.22E-06*
GO:0030595	*leukocyte chemotaxis*	*11*	*2.24E-06*
GO:0040011	*locomotion*	*30*	*2.49E-06*
GO:0060326	*cell chemotaxis*	*12*	*2.64E-06*
GO:0002690	*positive regulation of leukocyte chemotaxis*	*8*	*3.24E-06*
GO:0006928	*movement of cell or subcellular component*	*31*	*3.80E-06*
GO:0097529	*myeloid leukocyte migration*	*10*	*5.77E-06*
GO:0046849	*bone remodeling*	*8*	*6.00E-06*
GO:0046903	*secretion*	*24*	*7.94E-06*
GO:0009612	*response to mechanical stimulus*	*11*	*9.63E-06*
GO:0002688	*regulation of leukocyte chemotaxis*	*8*	*1.53E-05*

GO:0032940	*secretion by cell*	*22*	*1.62E-05*
GO:1901700	*response to oxygen-containing compound*	*27*	*2.25E-05*
GO:0032963	*collagen metabolic process*	*8*	*3.90E-05*
GO:0042127	*regulation of cell proliferation*	*27*	*4.03E-05*
GO:0044259	*multicellular organismal macromolecule metabolic process*	*8*	*5.06E-05*
GO:0010035	*response to inorganic substance*	*15*	*5.06E-05*
GO:0050921	*positive regulation of chemotaxis*	*8*	*5.78E-05*
GO:0048771	*tissue remodeling*	*9*	*6.85E-05*
GO:0002548	*monocyte chemotaxis*	*6*	*8.97E-05*
GO:0009607	*response to biotic stimulus*	*20*	*9.05E-05*
GO:0045087	*innate immune response*	*18*	*9.32E-05*
