# Supplementary material for: Wnt co-receptors Lrp5 and Lrp6 differentially mediate Wnt3a signaling in osteoblasts
Source: PLoS One. 2017 Nov 27;12(11):e0188264. doi: 10.1371/journal.pone.0188264 (PMC5703471; doi:10.1371/journal.pone.0188264)
Supplement: S8 Table — (PDF) [file pone.0188264.s009.pdf]

S8 Table. Top 50 enriched GO terms associated with genes differentially expressed in *Lrp6* ^{KO} osteoblasts compared to *Lrp6* $^{fl/fl}$ +TMX

Genes up-regulated in *Lrp6* ^{KO} Osteoblasts			
GO ID	***Enriched Biological Process***	***No. genes***	***FDR***
GO:0060337	type I interferon signaling pathway	12	6.44E-09
GO:0071357	cellular response to type I interferon	12	6.44E-09
GO:0034340	response to type I interferon	12	7.70E-09
GO:0045087	innate immune response	30	1.32E-08
GO:0006955	immune response	40	7.43E-08
GO:0006952	defense response	41	7.43E-08
GO:0009615	response to virus	19	1.24E-06
GO:0044419	interspecies interaction between organisms	26	1.24E-06
GO:0044403	symbiosis, encompassing mutualism through parasitism	26	1.24E-06
GO:0035456	response to interferon-beta	7	1.41E-06
GO:0051607	defense response to virus	17	1.41E-06
GO:0030198	extracellular matrix organization	17	1.99E-06
GO:0043062	extracellular structure organization	17	1.99E-06
GO:0034341	response to interferon-gamma	12	3.02E-06
GO:0002682	regulation of immune system process	35	5.10E-06
GO:0009607	response to biotic stimulus	28	5.93E-06
GO:0002252	immune effector process	25	8.22E-06
GO:0043207	response to external biotic stimulus	27	8.39E-06
GO:0051707	response to other organism	27	8.39E-06
GO:0032606	type I interferon production	10	1.03E-05
GO:0071345	cellular response to cytokine stimulus	22	2.04E-05
GO:0098542	defense response to other organism	20	2.85E-05
GO:0071346	cellular response to interferon-gamma	10	4.14E-05
GO:0034097	response to cytokine	23	5.44E-05
GO:1902988	neurofibrillary tangle assembly	3	7.40E-05
GO:1902996	regulation of neurofibrillary tangle assembly	3	7.40E-05
GO:0032479	regulation of type I interferon production	9	7.55E-05
GO:0035458	cellular response to interferon-beta	5	1.36E-04
GO:0032480	negative regulation of type I interferon production	6	1.66E-04
GO:0016032	viral process	21	1.66E-04
GO:0050776	regulation of immune response	23	1.84E-04
GO:0044764	multi-organism cellular process	21	2.07E-04
GO:0040011	locomotion	34	2.09E-04
GO:1902947	regulation of tau-protein kinase activity	3	2.19E-04
GO:0001501	skeletal system development	17	2.19E-04
GO:0031347	regulation of defense response	21	4.63E-04
GO:0080134	regulation of response to stress	31	5.59E-04
GO:0001816	cytokine production	19	5.67E-04
GO:0002684	positive regulation of immune system process	23	5.74E-04
GO:0006915	apoptotic process	35	6.19E-04

GO:0002697	regulation of immune effector process	15	7.28E-04
GO:0012501	programmed cell death	35	8.17E-04
GO:1902430	negative regulation of amyloid-beta formation	3	8.71E-04
GO:0051701	interaction with host	9	1.11E-03
GO:0019221	cytokine-mediated signaling pathway	16	1.22E-03
GO:0045088	regulation of innate immune response	13	1.22E-03
GO:0060338	regulation of type I interferon-mediated signaling pathway	5	1.42E-03
GO:0030574	collagen catabolic process	6	1.42E-03
GO:0001817	regulation of cytokine production	17	1.53E-03
GO:0032647	regulation of interferon-alpha production	4	1.99E-03

**Genes down-regulated in *Lrp6* ^{KO} Osteoblasts**			
GO ID	***Enriched Biological Process***	***No. genes***	***FDR***
GO:0030049	muscle filament sliding	13	4.73E-19
GO:0033275	actin-myosin filament sliding	13	4.73E-19
GO:0061061	muscle structure development	28	8.17E-19
GO:0042692	muscle cell differentiation	23	1.34E-17
GO:0003012	muscle system process	23	2.81E-17
GO:0051146	striated muscle cell differentiation	20	5.62E-17
GO:0006936	muscle contraction	21	8.95E-17
GO:0055001	muscle cell development	17	1.66E-16
GO:0070252	actin-mediated cell contraction	14	9.96E-16
GO:0055002	striated muscle cell development	16	1.03E-15
GO:0007517	muscle organ development	21	1.28E-15
GO:0014706	striated muscle tissue development	20	1.32E-14
GO:0030048	actin filament-based movement	14	1.32E-14
GO:0060537	muscle tissue development	20	2.85E-14
GO:0030239	myofibril assembly	11	1.16E-13
GO:0006941	striated muscle contraction	14	3.95E-13
GO:0045214	sarcomere organization	9	5.56E-12
GO:0031032	actomyosin structure organization	12	2.58E-10
GO:0030029	actin filament-based process	20	2.58E-10
GO:0048738	cardiac muscle tissue development	12	6.64E-09
GO:0060047	heart contraction	13	7.89E-09
GO:0003015	heart process	13	8.62E-09
GO:0048646	anatomical structure formation involved in morphogenesis	24	1.37E-08
GO:0043501	skeletal muscle adaptation	6	8.65E-08
GO:0060048	cardiac muscle contraction	9	1.29E-07
GO:0072359	circulatory system development	20	3.93E-07
GO:0072358	cardiovascular system development	20	3.93E-07
GO:0008015	blood circulation	15	4.26E-07
GO:0003013	circulatory system process	15	4.42E-07
GO:0010927	cellular component assembly involved in morphogenesis	12	7.15E-07
GO:0007507	heart development	15	8.90E-07

GO:0003009	skeletal muscle contraction	6	1.27E-06
GO:0014888	striated muscle adaptation	6	2.14E-06
GO:0014902	myotube differentiation	8	2.18E-06
GO:0006928	movement of cell or subcellular component	25	2.97E-06
GO:0045445	myoblast differentiation	7	3.53E-06
GO:0007519	skeletal muscle tissue development	9	3.84E-06
GO:0044057	regulation of system process	14	4.65E-06
GO:0050881	musculoskeletal movement	6	4.65E-06
GO:0050879	multicellular organismal movement	6	4.65E-06
GO:0030036	actin cytoskeleton organization	14	4.90E-06
GO:0060538	skeletal muscle organ development	9	5.28E-06
GO:0035051	cardiocyte differentiation	8	6.20E-06
GO:0045844	positive regulation of striated muscle tissue development	6	1.04E-05
GO:0048636	positive regulation of muscle organ development	6	1.04E-05
GO:0048747	muscle fiber development	6	1.09E-05
GO:1901863	positive regulation of muscle tissue development	6	1.09E-05
GO:0090257	regulation of muscle system process	9	1.09E-05
GO:0006937	regulation of muscle contraction	8	1.34E-05
GO:0009887	animal organ morphogenesis	18	1.67E-05
